# Advancing Anticancer Drug Discovery: Leveraging Metabolomics and Machine Learning for Mode of Action Prediction by Pattern Recognition

**DOI:** 10.1002/advs.202404085

**Published:** 2024-10-21

**Authors:** Mohamad Saoud, Jan Grau, Robert Rennert, Thomas Mueller, Mohammad Yousefi, Mehdi D. Davari, Bettina Hause, René Csuk, Luay Rashan, Ivo Grosse, Alain Tissier, Ludger A. Wessjohann, Gerd U. Balcke

**Affiliations:** ^1^ Leibniz Institute of Plant Biochemistry Dept. of Bioorganic Chemistry Weinberg 3 06120 Halle (Saale) Germany; ^2^ Martin Luther University Halle‐Wittenberg Institute of Computer Science 06120 Halle (Saale) Germany; ^3^ Martin Luther University Halle‐Wittenberg Medical Faculty University Clinic for Internal Medicine IV (Hematology/Oncology) 06120 Halle (Saale) Germany; ^4^ Leibniz Institute of Plant Biochemistry Dept. of Cell and Metabolic Biology Weinberg 3 06120 Halle (Saale) Germany; ^5^ Martin Luther University Halle‐Wittenberg Institute of Chemistry Department of Organic and Bioorganic Chemistry 06120 Halle (Saale) Germany; ^6^ Dhofar University Research Center Frankincense Biodiversity Unit Salalah 211 Oman

**Keywords:** cancer, drug discovery, machine learning, metabolomics, mode of action

## Abstract

A bottleneck in the development of new anti‐cancer drugs is the recognition of their mode of action (MoA). Metabolomics combined with machine learning allowed to predict MoAs of novel anti‐proliferative drug candidates, focusing on human prostate cancer cells (PC‐3). As proof of concept, 38 drugs are studied with known effects on 16 key processes of cancer metabolism, profiling low molecular weight intermediates of the central carbon and cellular energy metabolism (CCEM) by LC‐MS/MS. These metabolic patterns unveiled distinct MoAs, enabling accurate MoA predictions for novel agents by machine learning. The transferability of MoA predictions based on PC‐3 cell treatments is validated with two other cancer cell models, i.e., breast cancer and Ewing's sarcoma, and show that correct MoA predictions for alternative cancer cells are possible, but still at some expense of prediction quality. Furthermore, metabolic profiles of treated cells yield insights into intracellular processes, exemplified for drugs inducing different types of mitochondrial dysfunction. Specifically, it is predicted that pentacyclic triterpenes inhibit oxidative phosphorylation and affect phospholipid biosynthesis, as confirmed by respiration parameters, lipidomics, and molecular docking. Using biochemical insights from individual drug treatments, this approach offers new opportunities, including the optimization of combinatorial drug applications.

## Graphical abstract

This study explores the integration of metabolomics with machine learning to predict the mode of action (MoA) of anti‐proliferative drugs in cancer cells. Complex metabolite patterns of drug‐treated cells are trained by machine learning to classify the drug's MoA. Finally, the methodology is applied to predict the MoAs of novel anticancer agents, offering a promising tool during drug discovery and development.

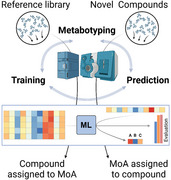

## 1 Introduction

Anti‐cancer drug discovery usually starts with in vitro viability assays using cancer cell line models, which identify cytotoxic chemicals with high anti‐proliferative capacity. At that early stage, knowledge regarding the mechanisms by which cell growth is inhibited is limited. Therefore, early characterization of the mechanism of action (MeA) or mode of action (MoA) of a drug has the potential to advance the drug development process.^[^ ^1^ ^]^ MeA refers to the specific biochemical interaction between a compound and its molecular targets, while MoA refers to the physiological effects caused by the compound of study. Metabolomics can help delineating MoAs by identifying metabolic changes in cells upon drug exposure, which are reflected in complex metabolic patterns (metabotypes). Prediction of the MoA for uncharacterized compounds is based on the hypothesis that drugs with similar targets will have similar effects on the metabolome.^[^ ^2^ ^]^ Machine learning (ML) can then be employed to predict the MoA(s) of a compound by using reference drugs with known MoA as training set, as has been recently demonstrated for antimicrobial compounds.^[^ ^2^, ^3^, ^4^ ^]^ While the focus of this study is on the MoA of potential anti‐cancer drugs, machine‐learning methods have previously been applied to understand the influence of perturbations on metabolic networks in the context of anti‐bacterial drugs and nanoparticles.^[^ ^5^, ^6^ ^]^ Notably, the machine learning methods applied show similarities to those considered in this study, namely k‐nearest neighbor classifiers and Random Forests.^[^ ^6^ ^]^

In cancer biology, such comprehensive studies of drug responses at the metabolites level are just beginning to emerge. Using prostate cancer cell lines, cell viability assays, and metabolomics, Lu et al. screened synergistic effects of a glutaminase inhibitor and a library of 292 anti‐cancer compounds.^[^ ^7^ ^]^ Lately, multi‐omics data of 54 cancer cell lines involving metabolomics analysis were used to correlate transcriptional regulation of tumor growth with metabolism.^[^ ^8^ ^]^ Anglada‐Girotto et al. combined CRISPRi and metabolomics to annotate drug libraries in *E.coli*.^[^ ^9^ ^]^ In implementing this combined approach, also human A549 alveolar cancer cells were exposed to 14 anti‐cancer drugs. In this side study, a strong accumulation of dUMP was observed only for those drugs targeting thymidylate synthase (TYMS), which was associated with a strong and selective similarity of metabolite patterns between genetically and chemically induced suppression of TYMS. This supports our hypothesis that using suitable analytical techniques, metabolomics can reveal crucial insights into the regulation of the central carbon and energy metabolism (CCEM).

The discovery that cancer cells exhibit abnormal regulation of central metabolic pathways led to the creation of highly specific tumor inhibitors.^[^ ^10^, ^11^, ^12^, ^13^, ^14^ ^]^ These inhibitors primarily target cancer cells by disrupting key processes such as glucose and glutamine uptake or metabolism, as well as blocking serine synthesis.^[^ ^15^, ^16^, ^17^ ^]^ Furthermore, in many cancers, pathological cell proliferation is associated with increased biosynthesis of nucleotides, acyl‐ and prenyl‐lipids. Therefore, metabolic inhibitors of cancer cell proliferation affect essential metabolic pathways such as *de novo* biosynthesis of NAD, the oxidative pentose phosphate pathway (OPP), fatty acid synthesis or the mevalonate pathway.^[^ ^18^, ^19^, ^20^ ^]^

Some chemotherapeutics such as taxol, vincristine or etoposide modify non‐metabolic targets like the cytoskeleton or affect DNA replication.^[^ ^21^, ^22^ ^]^ The PI3K/AKT/mTOR signaling pathway is another prominent target for cancer treatment since it controls the metabolic activity of key CCEM pathways.^[^ ^23^, ^24^ ^]^ Since these targets may, therefore, have indirect effects on the CCEM, it is crucial to investigate whether metabolic patterns derived from cell treatments with microtubule inhibitors, topoisomerase inhibitors, or PI3K/AKT/mTOR inhibitors accurately predict their MoAs.

In this study, we employed a multi‐targeted approach to analyze 188 hydrophilic metabolites of the central carbon and energy metabolism (CCEM).^[^ ^25^ ^]^ We sought to predict the MoA(s) for new anti‐proliferative compounds in the cancer cell model PC‐3 using a combination of machine learning and CCEM reference profiles derived from treatments with well‐known cytotoxins or anti‐cancer drugs. Two probabilistic prediction models were developed and are discussed in view of their ability to predict single or even complex MoAs. Additionally, the analysis of metabolite signatures enhances our comprehension of regulatory principles in the CCEM upon drug treatment, with a focus on mitochondrial inhibitors. To validate selected model predictions, we performed wet lab tests and enzyme docking simulations, using pentacyclic triterpenes as an example.

## 2 Results

### 2.1 Training of Metabolic Patterns Allows MoA Prediction

In order to establish a reference framework for metabolic responses in the central carbon and energy metabolism (CCEM), we utilized IC_50_ treatments for 48 h in the PC‐3 prostate cancer cell model using 38 reference compounds with known mechanisms of action (MeA) (Table S1, Supporting Information). We selected the set of 38 compounds based on their diverse drug targets that impede PC‐3 cell growth, sufficient MeA knowledge, and inclusion of at least two drugs for each MeA.^[^ ^11^, ^12^, ^13^, ^14^ ^]^ In order to ensure data comparability and reproducibility, we opted to utilize the individual IC_50_ drug concentrations predetermined for all treatments. This approach ensures that all cell samples are expected to be in a comparable state prior to metabolomics analysis, both in terms of growth phase and growth inhibition. In addition, the use of IC_50_ concentrations was a good compromise between lower concentrations, such as IC_20_, which might not sufficiently capture subtle metabolic changes, and higher concentrations, such as IC_80_, which could obscure results with patterns of apoptosis and necrosis while not providing the minimum required sample material.

A novel methodological approach to obtain the cellular metabolome, as unbiased as possible, was to harvest the cells by using high‐field ultrasound after carefully removing the dead cells, so that the live cells could be dispersed in situ in a cold quenching solution instead of trypsinizing or manually scraping them off^[^ ^26^, ^27^ ^]^ (Figure S1A, Supporting Information). We analyzed 188 hydrophilic metabolites from cell extracts, with 117 quantified reliably. These were normalized to ensure the sum of signals was proportional to cell counts (Figure S1B, Supporting Information), and then we assessed the similarity across all obtained metabolic profiles (Tables S2 and S3, Supporting Information).^[^ ^25^ ^]^

**Figure 1:**
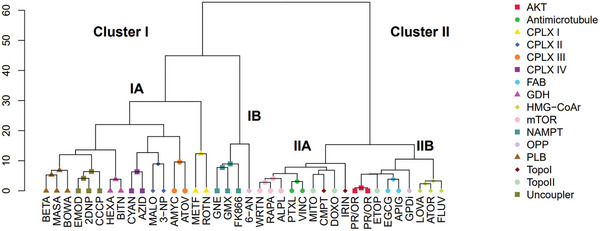
Hierarchical cluster analysis of metabolic patterns induced by 38 reference compounds inhibiting different molecular targets modulating the metabolism of prostate cancer cells (PC‐3). Aggregated leaves of hexuplicate experimental data are presented. Metabolic patterns of AKT inhibitors were so similar that individual replicates of the inhibitors PR and OR co‐clustered with each other. For this reason, we defined a mixed group “PR/OR”. A complete cluster analysis of all individual training samples is presented in Figure S2 (Supporting Information). Compounds: BETA – betulinic acid, MASA – maslinic acid, BOWA – boswellic acid, EMOD – emodin, 2DNP – 2,4‐dinitrophenol, CCCP – carbonyl cyanide chlorophenylhydrazone, HEXA – hexachlorophene, BITN – bithionol, CYAN – potassium cyanide, AZID – sodium azide, MALO – malonic acid, 3‐NP – 3‐nitropropionic acid, AMYC – antimycin A, ATOV – atovaquone, METF – metformin, ROTN – rotenone, GNE – GNE‐617, GMX – GMX1778, FK866 – FK866, 6‐AN – 6‐aminonicotinamide, WRTN – wortmannin, RAPA – rapamycin, ALPL – alpelisib, PTXL – paclitaxel, VINC – vincristin, MITO – mitoxantrone, CMPT – camptothecin, DOXO – doxorubicin, IRIN – irinotecan, PRFN (PR) – perifosine, ORID (OR) – oridonin, ETOP – etoposid, EGCG – epigallocatechin gallate, APIG – apigenin, GPDi – glucose‐6‐phosphate dehydrogenase inhibitor, LOVA – lovastatin, ATOR – atorvastatin, FLUV – fluvastatin. MoA: AKT – protein kinase B (AKT), Antimicrotubule, CPLX I – complex I, CPLX II – complex II, CPLX III – complex III, CPLX IV – complex IV, FAB – fatty acid biosynthesis, GDH – glutamate dehydrogenase, HMG‐CoAr – HMG‐CoA reductase, mTOR – PI3K/mTOR signaling, NAMPT – nicotinamide phosphoribosyltransferase, OPP – oxidative pentose phosphate pathway, PLB – phospholipid biosynthesis, TopoI – topoisomerase I, TopoII – topoisomerase II, Uncoupler – uncoupling of oxidative phosphorylation.

For the majority of MoA training groups (95%), hierarchical clustering of the metabolic profiles revealed co‐segregation of those training compounds addressing the same target (**Figure** **1**). Interestingly, the dendrogram identifies two main clusters: one harboring compounds affecting mitochondrial electron transport processes, oxidative phosphorylation, glutamate and NAD metabolism (cluster I); the second one comprising MoA patterns of other metabolic processes, some of them known to proceed in the cytosol (e.g., hydroxymethylglutaryl‐CoA reduction (HMG‐CoAr; EC 1.1.1.88), or fatty acid biosynthesis by fatty acid synthase (FASN, EC 2.3.1.85) (cluster II).^[^ ^19^, ^28^ ^]^ Other inhibitors assigned to this cluster are known to impair microtubule formation or degradation, to inhibit topoisomerases, or to modulate PI3K/AKT/mTOR signaling.

Only a few reference compounds in our analysis targeting the same MeA did not co‐cluster, for example, the inhibitors of the oxidative pentose phosphate pathway (OPP), 6‐AN (6‐aminonicotinamide) and G6PDi, both causing accumulation of 6‐phosphogluconate, the substrate of 6‐phosphogluconate dehydrogenase (6‐PGDH, EC 1.1.1.44) (Figure S3, Supporting Information). However, their drug‐induced metabolic patterns in PC‐3 showed different clustering behavior. 6‐AN's pattern co‐segregated with nicotinamide phosphoribosyltransferase (NAMPT, EC 2.4.2.12) inhibitors, while G6PDi's pattern showed higher similarity with protein kinase B (AKT, EC 2.7.11.1) and fatty acid biosynthesis (FAB) (Figure 1). Another interesting finding was that etoposide did not cluster with four other topoisomerase inhibitors tested, suggesting unique metabolic interactions resulting in a distinct metabotype.

#### 2.1.1 Inhibition of Mitochondrial Functions Produces Distinctive Metabotypes

Although cancer cells mainly produce ATP by enhancing glycolytic flux, they still require functional mitochondria,^[^ ^29^, ^30^, ^31^ ^]^ not least since oxidative phosphorylation (OXPHOS) is connected to seven ubiquinone (CoQ)‐dependent mitochondrial dehydrogenases with essential functions in CCEM.^[^ ^32^ ^]^ Consequently, targeting different stages of OXPHOS has specific effects on various metabolic processes linked to these CoQ‐dependent mitochondrial dehydrogenases.

**Figure 2:**
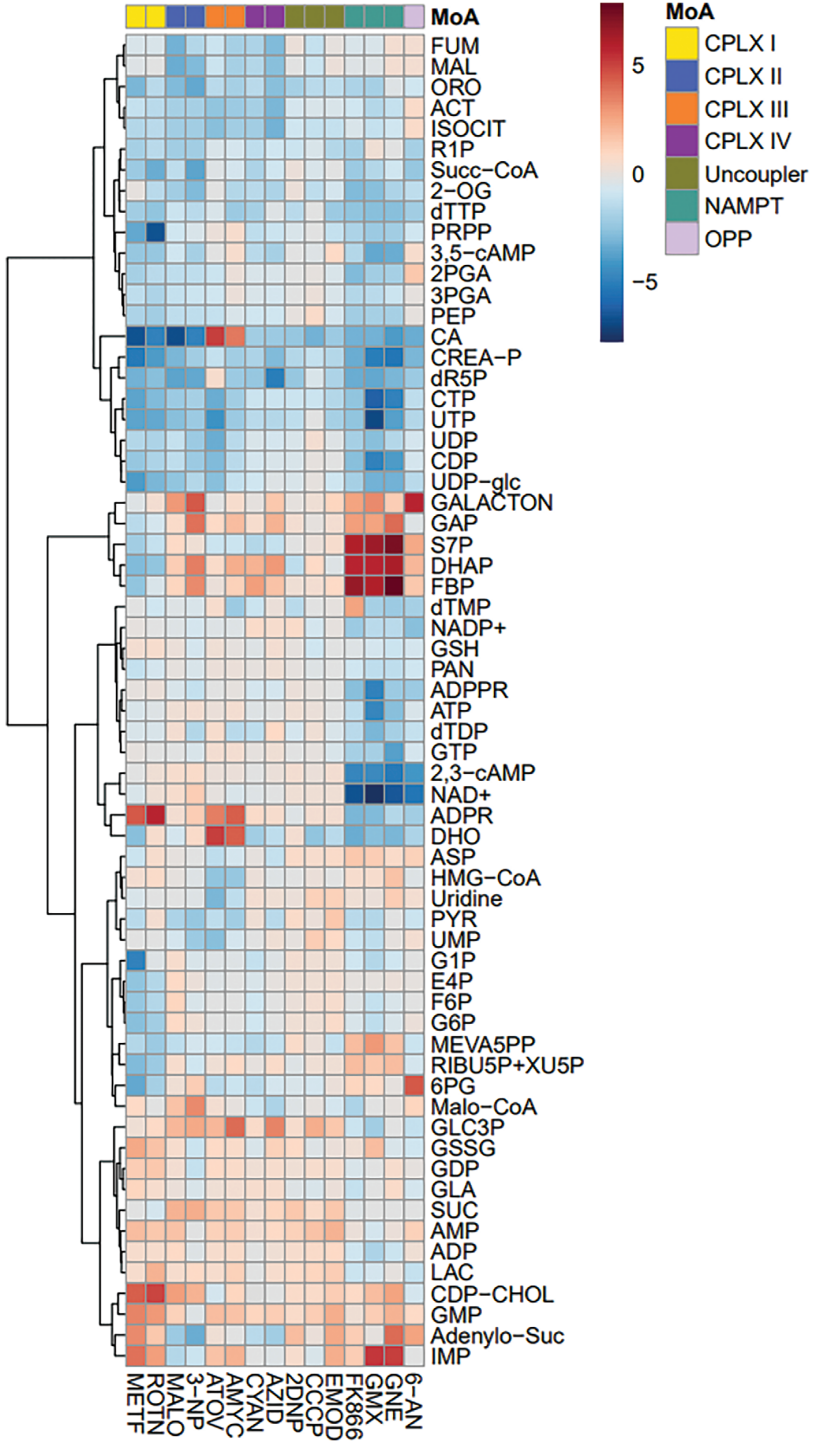
Relative abundance of selected CCEM intermediates after OXPHOS inhibition of the complexes I‐IV, application of uncouplers, inhibition of nicotinamide phosphoribosyltransferase (NAMPT), and the oxidative pentose phosphate pathway. Data depict average log_2_‐fold changes of cell number‐normalized peak areas obtained after 48 h drug treatment (*n* = 6) relative to vehicle control (*n* = 6). Legends of MoA and compound labels are given in Figure 1 and Tables S1 and S2 (Supporting Information).

**Figure 3:**
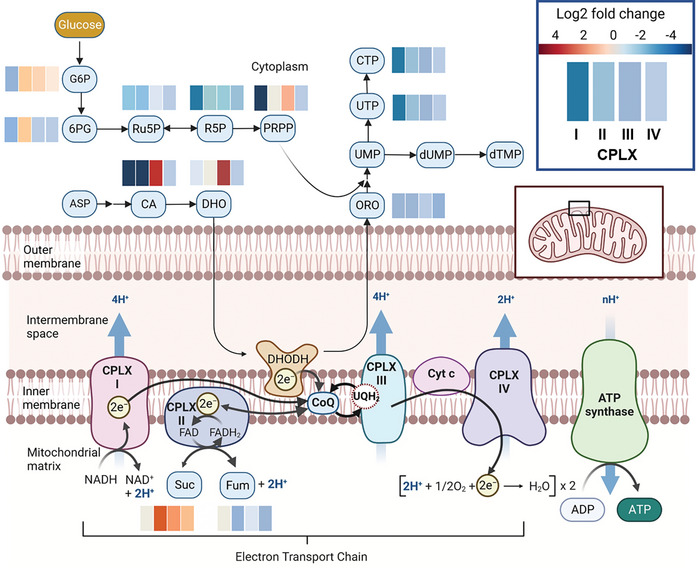
Relative abundance of selected intermediates from the pentose phosphate pathway, TCA cycle and pyrimidine biosynthesis in response to CPLX I‐IV inhibition. The heatmap displays the log₂ fold changes of these metabolites compared to untreated controls across treatments with inhibitors of CPLX I, II, III, and IV, respectively (from left to right). The mitochondrial coenzyme Q junction links pyrimidine nucleotide biosynthesis to the mitochondrial ETC. In order to fuel ATP formation, OXPHOS generates a proton gradient across the inner mitochondrial membrane through the electron transfer chain (ETC), which involves four protein complexes (CPLX I‐CPLX IV). Both CPLX I and CPLX II transfer electrons to ubiquinone (coenzyme Q, CoQ) in the inner mitochondrial membrane resulting in ubiquinol (UQH2) reconstitution. By contrast, CPLX III is responsible for the reoxidation of ubiquinol to ubiquinone, which is important not only for the function of CPLX I and CPLX II. CPLX IV then transfers the electrons to molecular oxygen. Metabolite abbreviations are given in Table S2 ((Supporting Information)). This graph was created with BioRender.com.

Initially, we observed that OXPHOS inhibitors lead to specific patterns of metabolites that are categorized into cluster I (Figure 1). These patterns further subdivide into distinct sub‐clusters depending on which complex (CPLX) of the respiratory chain is inhibited. When CPLX I to IV are inhibited, a consistent pattern becomes evident in certain TCA cycle metabolites. Specifically, levels of cis‐aconitate (ACT), isocitrate (ISOCIT), and succinyl‐CoA (SUC‐CoA) are depleted (**Figure** **2**). This pattern is not observed in the case of uncouplers. Remarkably, specific to CPLX I inhibition, neither the accumulation of succinate (SUC) nor the strong depletions of fumarate (FUM) and malate (MAL) that are typical of CPLX II‐IV inhibition is observed (**Figure** **3**).

In the cytosol, OXPHOS inhibitors and uncouplers generally induce the accumulation of glycerol 3‐phosphate (Glc3P) (Figure 2). However, just as observed in the TCA cycle, sugar metabolism also displays distinct characteristics when CPLX I inhibition is compared to CPLX II‐IV inhibition. Notably, dihydroxyacetone phosphate (DHAP) showed an up to eight‐fold depletion after CPLX I inhibition but was increased after inhibition of CPLX II‐IV (Figure S4, Supporting Information). Elevating Glc3P synthesis could potentially rescue complex I deficiency by the provision of reducing equivalents to CoQ by the Glc3P shuttle.^[^ ^33^ ^]^ Nevertheless, both CPLX I inhibitor treatments, rotenone, and metformin, consistently lead to the depletion of numerous glycolytic and pentose phosphate pathway intermediates, inferring suppressed sugar metabolism in PC‐3 cells and limited substrate availability for the Gl3P shuttle (Figure 2).

In contrast, inhibiting CPLX II‐IV leads to a different glycolytic pattern compared to inhibiting CPLX I, with fructose 1,6‐bisphosphate (FBP), DHAP, and Glc3P accumulating while lower glycolysis metabolites are depleted (Figure 2). This suggests that the glycerol phosphate shuttle responds differently to severe energy depletion in these two cases. It is worth noting that CPLX I contributes to 40% of ATP synthesis through the proton motive force, as reported by Wang et al.^[^ ^14^ ^]^ Although, ATP levels did not decrease significantly after inhibiting OXPHOS, the depletion of energy‐conserving phosphocreatine was much more pronounced after CPLX I inhibition (up to 196‐fold decline) compared to CII‐CPLX IV inhibition (five to nine‐fold decline) (Figure S3, Supporting Information).

Moreover, pyrimidines showed reduced concentrations when OXPHOS was inhibited at CPLX I‐IV but not when inhibited with uncouplers (Figure 2). Pyrimidine biosynthesis in mammals starts with CAD, a large trifunctional enzyme (composed of carbamoyl phosphate synthetase (CPS2), aspartate transcarbamoylase, and dihydroorotase). The impact of different modes of OXPHOS inhibition on CAD‐associated metabolites revealed discernible differences between CPLX III and the other respiratory complexes (Figure 3). For instance, inhibition of CPLX I and CPLX II led to a significant decrease (by 33 to 132‐fold) in carbamoylaspartate (CA), while inhibition of CPLX III resulted in 13–45 times higher levels than in control cells. Similarly, dihydroorotate (DHO), the product of the third step catalyzed by CAD, accumulated strongly (20‐36 fold) only after CPLX III inhibition. The following biosynthetic enzyme, dihydroorotate dehydrogenase (DHODH, EC 1.3.1.14), located in the mitochondrial membrane, oxidizes dihydroorotate to orotate (ORO) in a CoQ‐dependent manner.^[^ ^31^, ^32^ ^]^ Interestingly, OXPHOS inhibition generally caused a general decrease in orotate levels despite the contrasting CA and DHO patterns.

Moreover, in cancer cells, glutamine/glutamate catabolism is a major source to fuel the citric acid cycle with carbon and to provide energy via OXPHOS. Glutamate dehydrogenase (GDH) is a mitochondrial enzyme, which catalyzes the reversible conversion of glutamate to the TCA cycle intermediate 2‐oxoglutarate and ammonium. Consequently, co‐segregation of metabolic patterns after GDH inhibition as compared with the inhibition of other mitochondrial functions in sub‐cluster IA makes sense (Figure 1).

Metabolic patterns of treatments with pentacyclic triterpenes likewise emerge in cluster I. Several pentacyclic triterpene acids have been extensively studied for their anti‐cancer properties, including inhibition of cell proliferation and apoptosis induction.^[^ ^34^ ^]^ They target various genes and pathways such as Bcl‐2, NF‐kB, and PI3K/Akt/mTOR, as reviewed by Petrenko et al.^[^ ^35^ ^]^ Indication that such triterpenoids also affect phospholipid biosynthesis (PLB) has been provided by micelle models mimicking mitochondrial membranes, where membrane rigidification and increased membrane permeabilization were induced in a dose‐dependent manner.^[^ ^36^ ^]^ A recent study investigated the MeA of ursolic acid in human prostate cancer models and found an accumulation of intermediates of PLB.^[^ ^37^ ^]^ Nevertheless, the precise impact of pentacyclic triterpenoids on cancer cell metabolism remains hitherto only partially understood.

To further investigate these effects at the metabolic level we assessed the metabotypes of 11‐keto‐β‐boswellic acid (BOWA), maslinic acid (MASA), and betulinic acid (BETA) in PC‐3 cells. Our data suggests that pentacyclic triterpenoids affect mitochondrial metabolism, exhibiting metabolic patterns akin to uncouplers and GDH inhibition (Figure 1). Despite sharing some characteristics of OXPHOS inhibition in their metabolic profiles, they differ from conventional OXPHOS inhibitors, which are further investigated below.

#### 2.1.2 Inhibition of NAD Salvage Leads to Severe ATP Starvation

In cancer cells, NAD is mainly synthesized from nicotinamide via NAD salvage.^[^ ^38^ ^]^ NAMPT is the rate‐limiting enzyme in the NAD salvage and is therefore a popular therapeutic target.^[^ ^39^ ^]^ Expectedly, NAMPT inhibition results in a metabolic pattern, which is characterized by strong depletion of NAD and NADP but also of their cleavage products ADP‐ribose (ADPR) and ADP‐ribose 2′‐phosphate (ADPPR) (Figure 2). NAD(P) depletion, however, has several consequences leading to a specific metabolic pattern (Figure 1, sub‐cluster IB), one of them is a strong depletion in ATP (Figure 2; Figure S3, Supporting Information). Despite significant cell growth inhibition throughout these experiments, we noticed consistent intracellular levels of three pivotal metabolites within the CCEM. Across all investigated MoAs, except for NAMPT inhibition affecting ATP, a balanced state is maintained for three metabolites: ATP, acetyl‐CoA (AcCoA), and citrate (CIT) (Figure S3, Supporting Information). Intracellular ATP homeostasis is normally maintained under moderate energy deprivation by activation of creatine kinase (EC 2.7.3.2) and adenylate kinase (EC 2.7.4.3). Consistently, for MoAs affecting energy metabolism, we observed reduced levels of energy‐storing phosphocreatine (CREA‐P), and changes in the levels of inosine monophosphate (IMP) and adenylosuccinate (Adenylo‐Suc), the latter two being involved in the purine nucleotide cycle that balances the equilibrium of adenylate kinase (Figure S3, Supporting Information).

Limited availability of NAD also impairs the glycolytic ATP generation in cancer cells since NAD is a required coenzyme of the enzyme glyceraldehyde‐3‐phosphate dehydrogenase which fosters the diversion of the carbon flow into upper glycolysis and pentose phosphate pathway (PPP).^[^ ^20^, ^40^ ^]^ Accordingly, the metabolic patterns of three NAMPT inhibitors consistently show very strong accumulation of fructose 1,6‐bisphosphate (FBP) and triose phosphates, accompanied by a depletion of intermediates of the lower glycolysis such as 3‐phosphoglyceric acid (3‐PGA), phosphoenolpyruvate (PEP) and pyruvate (PYR) (Figure 2). Lack of NAD(P) also affects intermediate levels of the non‐oxidative pentose phosphate pathway, resulting in increased levels of pentose phosphates and sedoheptulose‐7‐phosphate (S7P), whereas 6‐phosphogluconate (6‐PG) levels of the OPP remain unaffected (Figure 2; Figure S3, Supporting Information). This discriminates NAMPT inhibitors from OXPHOS inhibitors, which do not accumulate pentose or heptulose phosphates, and from direct OPP inhibitors, which strongly accumulate 6‐PG.

However, the metabolic patterns of both OPP inhibitors tested diverged, with only 6‐AN sharing similarities with the metabolic patterns detected after NAMPT inhibition. Notably, the second OPP inhibitor tested, G6PDi, was previously described to inhibit only glucose 6‐phosphate dehydrogenase (G6PDH, EC 1.1.1.49), while 6‐AN is known to inhibit also the second line of NADPH producing enzymes in the OPP, 6‐PGDH.^[^ ^41^, ^42^ ^]^ Until today, it has not been described whether 6‐AN acts as nicotinamide analog in NAD salvage via NAMPT converting 6‐AN to 6‐amino‐NAD. However, it is known that 6‐amino‐NAD(P) are potent inhibitors of metabolic processes requiring NAD(P) such as glycolysis and OPP.^[^ ^43^ ^]^ Therefore, strong commonalities between NAMPT inhibitors and 6‐AN indicate that 6‐amino‐NAD might act as a NAMPT inhibitor as well.

In summary, inhibitors of NAD biosynthesis and OXPHOS inhibitors share great similarities in their metabolic patterns. Since both have a strong influence on cellular energy metabolism, their patterns are also grouped together in a common cluster (cluster I, Figure 1).

#### 2.1.3 Metabolite Patterns Specific for Non‐Mitochondrial Targets

In Figure 1, similarity cluster II comprises MoAs that are not directly associated with mitochondrial targets such as microtubules, fatty acid biosynthesis (FAB) or hydroxymethylglutaryl CoA reductase (HMG‐CoAr). Despite the absence of a direct impact on energy metabolism, compounds in cluster II are grouped according to their MoAs, with patterns that reflect specific influence on central carbon and energy metabolism (CCEM) (Figure S5, Supporting Information).

Cluster analysis further unveiled two sub‐clusters, IIA and IIB. Surprisingly, the metabolic patterns of mTOR inhibitors and AKT inhibitors, both targeting the PI3K/AKT/mTOR pathway, did not cluster in the same sub‐cluster. Even though both AKT and mTOR inhibitors influence the same signaling pathway, their distinct patterns led us to employ orthogonal partial least square analysis (oPLS‐DA) for differentiation. This analysis effectively distinguishes mTOR inhibitors (rapamycin, wortmannin, alpelisib) from AKT inhibitors (oridonine, perifosine) (Figure S6, Supporting Information). Notably, the most significant differences between these MoAs arise from lower levels of 6‐PG, GLc3P, and thymidylates with mTOR inhibitors compared to AKT inhibitors. Conversely, mTOR inhibitors exhibit higher levels of ATP and aspartate compared to AKT inhibitors.

Except of etoposide (ETOP), topoisomerase I and II inhibitors induce a specific metabotype in PC‐3 cells, where several glycolytic intermediates (2‐PGA, 3‐PGA, FPB) and pentose phosphate pathway intermediates (6‐PG, R5P, Ru5P + XU5P, and S7P) were depleted compared to the untreated control. Although Jaccard clustering was unable to further distinguish between the two topoisomerase subtypes, we observed an accumulation of several coenzyme A esters with the topoisomerase I inhibitors camptothecin and irinotecan, which is absent when using topoisomerase II inhibitors mitoxantrone and doxorubicin (Figure S5, Supporting Information, panels A–D).

Furthermore, three statins – lovastatin (LOVA), fluvastatin (FLUV), and atorvastatin (ATOR) – produced a specific metabotype in PC‐3 cells with HMG‐CoA accumulation and reduction of mevalonate 5‐pyrophosphate, which is downstream of HMG‐CoA reductase, as expected (Figure S5, Supporting Information, panels E–G). This pattern comprises a variety of yet unknown metabolic consequences, primarily the inhibition of thymidylate synthesis (lower dTMP and dTTP) accompanied by decreased levels of CA, DHO, and ORO in the early pyrimidine biosynthesis. Further investigation of this link between mevalonate pathway inhibition and pyrimidine biosynthesis would be crucial, not least because the mevalonate pathway is the source of ubiquinone, an essential molecule for pyrimidine biosynthesis, and the electron transport chain.

In summary, in cluster I, co‐segregated metabolic patterns strongly suggest mitochondrial dysfunction, distinguishing between OXPHOS inhibition and NAD metabolism inhibition. These differ distinctly from MoA patterns in cluster II, which aid in identifying MoAs affecting non‐mitochondrial targets or indirectly influencing metabolism.

#### 2.1.4 Evaluation of Different Machine Learning (ML) Approaches to Predict Metabolic Patterns

We apply machine learning with the goal of predicting the MoA of new drugs based on their normalized CCEM profile. Since training data (i.e., CCEM profiles of drugs with known MoA) are of high quality but rather sparse (2‐3 drugs per MoA), we needed to resort to methods that can accomplish classification under these constrains (Tables S1 and S3, Supporting Information). Specifically, we used Random Forests for classification as they provide reliable results with sparse training data. Additionally, Lasso regression was used to explain an unclassified CCEM profile based on a limited selection of classified profiles in the training set. Random Forests are specifically designed for classification problems and aim to avoid overfitting by repeated sampling from the variables considered in decision trees and bootstrapping on the training samples. The Lasso‐based approach fits coefficients for the CCEM profiles of all drugs in the training set to explain the CCEM profile of a test drug (response variable), while regularization yields coefficients different from zero only for those training CCEM profiles that contribute to the fit substantially. The MoAs of training CCEM profiles that obtain large coefficients in the fit and, hence, contribute the most to explaining the test CCEM profile are considered for predicting the MoA of the test drug. As different drugs of the training set may obtain coefficients different from zero, the Lasso‐based approach is expected to handle intermediate/mixed MoAs more appropriately. To establish a baseline, we also incorporated a simple k‐nearest neighbor classifier based on Euclidean distance to train CCEM profiles.

Limitations of the quantity of high‐quality training data available render partitioning into dedicated training and test sets for evaluating the alternative approaches unreasonable. To establish a performance evaluation across all drugs and associated MoAs, nonetheless, we follow a leave‐one‐out cross‐validation strategy. Here, the data are partitioned such that all replicates of one drug (test partition) excluded from the training set, the respective method is trained on the CCEM profiles of the remaining drugs in the training set, and the MoA of the individual replicates of the left‐out drug is predicted. By excluding all replicates of the test drug from the training set, we avoid data leakage in the same manner as for dedicated training and test sets (Tables S4 and Data S1, Supporting Information).

**Figure 4:**
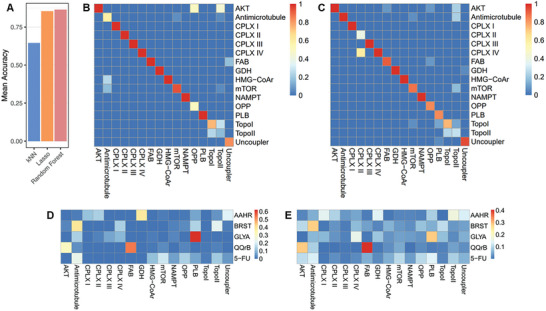
Performance evaluation and MoA prediction by machine learning approaches. A) Mean accuracy of k‐nearest neighbor classifier (kNN), Lasso‐based approach (Lasso), and Random Forest classifier (Random Forest) in leave‐one‐out cross‐validation over the drugs in the training set. B,C) Mean accuracy per MoA, where entries on the main diagonal correspond to correct classification and off‐diagonal elements indicate alternative MoA (row), B‐Lasso, C‐Random Forest. D,E) Normalized prediction scores for the MoAs of four cytotoxic compounds obtained by D‐Lasso‐, and E‐Random Forest classifier. An alternative visualization of the prediction results is provided in Figure S12 (Supporting Information), which shows the fraction of replicates assigned to a specific MoA according to the largest prediction score. MoA abbreviations are listed in Figure 1, GLYA – glycyrrhetinic acid, AAHR – asiatic acid homopiperazinyl rhodamine B conjugate, BRST – Breastin (patented *Nerium oleander* cold water extract), QQrB – cucurbitacin B. The R source code to generate Figure 4A–C is provided in Data S1 (Supporting Information).

Initially, prediction accuracy (classification rates) was averaged over all drugs sharing the same MoA and then across all MoAs. We found that the baseline model (k‐nearest neighbor) achieved a mean accuracy of 0.655, while the Lasso‐based approach and the Random Forest yielded substantially improved mean accuracy values of 0.854 and 0.864, respectively (**Figure** **4**A; Table S4 and Data S1, Supporting Information). Hence, we consider the Lasso‐based and the Random Forest approaches similarly suited for predicting MoAs of novel drug candidates.

Subsequently, the predictions of both approaches were evaluated on the level of MoAs to achieve a more fine‐grained picture of prediction performance. We found notable differences as to the prediction accuracy of individual MoAs (Figure 4B,C). Certain MoAs, such as CPLX I, III, and IV, GDH, HMG‐CoAr, and NAMPT, can be well predicted using either approach. Other MoAs (OPP, TopI, TopoII, Uncoupler) show reduced prediction performance by either approach, although to a varying degree. Finally, antimicrotubule, CPLX II, and PLB inhibitors can be classified almost perfectly by only one of the two approaches. Misclassification can be considered from two perspectives. First, replicate CCEM profiles of drugs from a certain MoA (e.g., TopoII) may be classified into multiple different MoAs. Second, some MoAs (e.g., AKT with Lasso, CPLX IV with Random Forest) co‐classify with other MoAs, which might be explained by the lack of unique metabolic patterns that allow discrimination between these MoAs. Finally, not all effects of a training inhibitor (“drug”) may be known yet for the cell line used.

### 2.2 ML Predicts MoAs of Novel Cytotoxic Compounds with Anti‐Cancer Potential

Next, both ML approaches were applied to predict the MoAs of 5‐fluorouracil (5‐FU), one semi‐synthetic, and three plant‐derived drug candidates (Table S1, Supporting Information). The novel compound AAHR, composed of triterpene asiatic acid and rhodamine B, has demonstrated robust cytotoxicity, and the capacity to conquer drug resistance in human preclinical tumor models.^[^ ^44^ ^]^ In contrast to glycyrrhetinic acid (GLYA, a pure pentacyclic triterpenoid acid derived from licorice roots), AAHR has a higher toxicity toward PC‐3 prostate cancer cells (Table S1, Supporting Information). Breastin (BRST) is a defined cold‐water extract from leaves of *Nerium oleander*. Phytochemical characterization revealed several monoglycosidic cardenolides as major constituents.^[^ ^45^ ^]^ Cucurbitacin B (QQrB) is a highly toxic tetracyclic triterpene drug derived from Cucurbitaceae plants (Table S1, Supporting Information).

#### 2.2.1 The Triterpene‐Rhodamine Conjugate AAHR is a Potent OXPHOS Inhibitor

ML approaches suggest that AAHR inhibits multiple targets, of which four out of five point to the inhibition of mitochondrial processes (Figure 4D,E; Figure S7, Supporting Information). Wet lab studies using lipid profiling, Seahorse cell analysis, and CoQ measurements confirmed this prediction showing that AAHR results in acylcarnitine accumulation, reduced mitochondrial respiration and ATP formation, and depletion of cellular CoQ levels (Figures S8,S9A, and S10 and Table S5, Supporting Information). In addition, confocal laser scanning microscopy revealed co‐localization of AAHR with the mitochondrial membrane in PC‐3 cells stained with MitoTracker Green (86% fluorescence co‐localization) (Figure S11, Supporting Information). To understand precisely how AAHR inhibits mitochondrial respiration, we modified the Seahorse assay by replacing individual OXPHOS inhibitors with AAHR (Figure S9B, Supporting Information). Based on the pattern of oxygen consumption rate (OCR) and extracellular acidification rate (ECAR), AAHR does not act as an uncoupling agent or complex V inhibitor. Rather, it inhibits mitochondrial respiration by targeting complex I‐III and/or by an indirect MoA affecting cell respiration.

#### 2.2.2 Pentacyclic Triterpenes are Potential Modulators of Phospholipid Biosynthesis (PLB)

Both ML models predicted that the MoA of GLYA involves modulation of PLB (Figure 4D,E), which is consistently identified as the most probable MoA of GLYA across all six replicates (Figure S12, Supporting Information). This prediction was supported by a strong accumulation of CDP‐choline and phosphoethanolamine (PEA) with several pentacyclic triterpenes, such as BETA (Figure S5, Supporting Information, panel H). However, CDP‐choline accumulation was not observed for all training compounds and neither for GLYA treatments. Nonetheless, the prediction based on the “PLB”‐metabotype is characteristic.

Accumulation of CDP‐choline is expected when choline phosphotransferase (CPT1, EC 2.7.8.1) or choline/ethanolamine phosphotransferase (CEPT1, EC 2.7.8.2) is inhibited in the Kennedy pathway. To further investigate this issue, we performed in silico docking of pentacyclic triterpenes and the natural substrate CDP‐choline on CEPT1 as well as CPT1 and found that pentacyclic triterpenoids could indeed be strong competitive inhibitors of both enzymes (Figure S12 and Table S6, Supporting Information).

Therefore, we assumed that administration of pentacyclic triterpenoids and AAHR would reduce cellular concentrations of intact phosphatidylcholines (PC) and investigated this by performing lipidomics. Instead of a general reduction, we found individually altered PC levels, reflecting large changes in the global membrane lipid composition after BETA and AAHR treatment (Figure S8, Supporting Information). Among the lipid groups showing the highest alterations were PC and PE as well as sphingomyelins (SM), which derive from PC metabolism. Interestingly, BETA and AAHR treatment induced the formation of lyso‐phosphatidylethanolamines (LPE) and lyso‐PC (LPC), respectively, indicating membrane destabilization and exposure of apoptosis‐inducing “eat‐me signals”.^[^ ^46^ ^]^

To further corroborate that triterpenoic acids inhibit PLB at the enzymatic step of CPT1, we labeled PC‐3 cells by replacing glucose in the cell culture medium with U^13^C‐glucose and concomitantly added BETA in the treatments over 48 h, whereas in controls no betulinic acid was added. Fractional enrichment clearly demonstrates that less ^13^C carbon is incorporated into the three most abundant PC when BETA is present (Figure S13, Supporting Information).

Because mitochondrial membranes account for a high proportion of all PC and PE, changes in the composition of both lipid groups could particularly be related to mitochondrial dysfunction.^[^ ^47^ ^]^ Mitochondrial dysfunction is strongly supported by the profiles of free fatty acids (FA), acylcarnitines (CAR), and triacylglycerides (TG), all of which were found to be increased after treatment with BETA and AAHR. These compounds are involved in energy replenishment in mitochondria, and their accumulation suggests impaired OXPHOS.^[^ ^48^ ^]^ Another indication of impaired OXPHOS is the strong decrease of CoQ in PC‐3 cells after BETA treatment (Figure S10, Supporting Information).

However, for GLYA and AAHR, both ML models predict additional modes of action besides PLB (Figure 4D,E). For AAHR, OXPHOS inhibition clearly predominates among ML‐based model predictions, whereas both models predict OXPHOS inhibition as a side effect for pure pentacyclic triterpenoic acids (Figure 4D,E).

#### 2.2.3 Breastin Impairs Microtubule Formation

For Breastin both ML approaches agree and assign the largest scores to the antimicrotubule MoA with further considerable scores for CPLX IV, PLB, and TopoII in case of Lasso and non‐zero scores for multiple MoA in case of the Random Forest (Figure 4D,E). Recently, in a comparative analysis with 153 anticancer agents with known MoA on 74 tumor cell lines of an Oncotest panel, Rashan et al. showed frequent correlations of Breastin to mitosis‐inhibiting and DNA damaging drugs.^[^ ^49^ ^]^ Consequently, the antimicrotubular activity of Breastin was further explored using tubulin‐GFP‐transfected U2OS cells. Confocal microscopy verified that Breastin acts as a tubulin‐depolymerizing agent, displaying behavior like paclitaxel, thus confirming the machine learning prediction made here.^[^ ^49^ ^]^

#### 2.2.4 Cucurbitacin B (QQrB) Inhibits Lipogenesis

Both ML models assigned QQrB to the MoA of fatty acid biosynthesis (FAB) with the highest score (Figure 4D,E), also considering predictions per replicate (Figure S12, Supporting Information). Cancer cells have various ways of maintaining their fatty acid levels, including increased de novo FAB.^[^ ^50^ ^]^ ATP citrate lyase (ACLY, EC 2.3.3.8), acetyl‐CoA carboxylase (ACC, EC 6.4.1.2), and fatty acid synthase (FASN, EC 2.3.1.85) are the key enzymes responsible for lipogenesis via FAB and are targeted by anti‐proliferative therapies.^[^ ^51^ ^]^ FAB inhibitors like epigallocatechin gallate (EGCG) and apigenin (APIG)impede lipogenesis at the FASN stage.^[^ ^52^, ^53^ ^]^ Similarly, QQrB inhibits ACLY, which produces a comparable metabolite profile to EGCG and APIG.^[^ ^54^ ^]^

#### 2.2.5 The MoA of 5‐Fluorouracil (5‐FU) Cannot be Predicted

While 5‐FU, a recognized TYMS inhibitor, displays a distinct response pattern with depletion of thymidinylates and elevated folate levels (Figure S5i, Supporting Information), this mode of action was not included in the training process. By concept, classification approaches cannot predict classes that are not represented in the training set. The lasso‐based approach is partly capable of interpolating between MoAs present in the training data, but nonetheless cannot predict MoAs beyond the training space. Consequently, neither Lasso nor random forest can accurately predict the MoA of 5‐FU. Both ML methods show low prediction scores for numerous MoAs (Figure 4D,E), however, allowing us to identify such cases based on prediction outcomes for further extension and improvement of our methodology.

### 2.3 Experimental Validation and Transferability of MoA Predictions to Other Cancer Cell Models

To assess the general applicability of the methodology also to other cancer cells beyond the PC‐3 prostate cell line, we conducted similar metabotyping experiments with two additional cell lines: MCF‐7 hormone receptor‐positive breast cancer cells and MHH‐ES‐1 Ewing's sarcoma cells. Specifically, we examined the effects of four compounds: atorvastatin (ATOV) and lovastatin (LOVA), both inhibitors of HMG‐CoA reductase (HMG‐CoAr), and bithionol (BITN) and hexachlorophorane (HEXA), both inhibitors of glutamate dehydrogenase (GDH). Utilizing cluster analysis and partial least squares discriminant analysis (PLS‐DA) to classify the MoAs, we observed that the metabolite profiles after drug treatment exhibited significant similarities across the three cancer cell models (Figure S14A,B, Supporting Information). Moreover, variable importance in the projection (VIP) metabolites, contributing most strongly to the separation of both MoAs, showed consistent regulation independent of the cancer cell model (Figure S14C,D, Supporting Information). Finally, we attempted to predict the MoAs GDH and HMG‐CoAr in both additional cell lines using PC‐3 data as a training set by using both ML approaches. While the predictions of the Random Forest classifier appear to suffer from cell line‐specific base profiles, the Lasso‐based approach successfully predicts the correct MoA for three out of four drugs and yields a widely consistent prediction across replicates for HEXA, ATOR, and LOVA (Figure S14E, Supporting Information).

## 3 Discussion

Our results in PC‐3 prostate cancer cells clearly show that the targeted UPLC‐MS/MS analysis of only 117 metabolites of CCEM allows a reliable assignment of one or even several concomitant MoAs through which a drug (candidate) acts on cancer cells, which in some cases also enables mechanistic insights. Although the approach has a somewhat lower throughput than solely HR‐MS‐based rapid screenings,^[^ ^3^, ^7^, ^55^ ^]^ it avoids misclassification caused by poorly annotated and artefactual signals and allows discrimination of important isobaric intermediates.^[^ ^56^ ^]^

Despite having a small training dataset, we could demonstrate the predictability of the MoA of new drug candidates. Overall, the Random Forest approach resulted in more evenly distributed prediction scores across MoAs compared to the Lasso‐based approach, which usually decides on one or a few MoAs. Because each decision tree in the Random Forest must decide on exactly one MoA this can lead to different predictions if the MoA of the drug does not match exactly with the training drugs. In contrast, the Lasso regression aims to explain the observed CCEM profile of the drug by a small subset of CCEM profiles, leading to coefficients of many MoAs being pushed toward zero. Thus, the Lasso‐based approach can better represent mixtures of MoAs because it considers additional MoAs as partial contribution(s) (different from zero) to improve the overall regression. In predictions across cell lines, the Lasso‐based approach also shows to be robust with regard to cell line‐specific base profiles and predicts the correct MoA for three out of four tested drugs.

When analyzing the transferability of the prediction method to other cancer cell lines, we found characteristic metabolic patterns associated with individual MoAs to be conserved, e.g. HMG‐CoA accumulates in all cell models after HMG‐CoA reductase inhibition. Nevertheless, and not surprisingly, correct MoA prediction based on PC‐3 training data is associated with lower predictive reliability in the two other cancer cell models investigated. Past analyses of the metabolomes in up to 928 cancer cell lines revealed significant variations in their basal metabolite profiles, primarily stemming from distinct enzymatic regulations within the CCEM. These variations are, in part, a result of epigenetic effects or of genetic mutations specific to the respective cell lines.^[^ ^55^, ^57^ ^]^ All three cancer cell models under exemplary investigation are known to carry specific gene mutations in various oncogenes affecting the CCEM (Figure S15, Supporting Information). The impact of individual mutations or epigenetic alterations on drug response in these models remains unknown. Thus, conducting comprehensive studies with additional cancer cell models, large drug libraries and machine learning will be crucial to understand the effects of individual mutations and drug treatments on metabolism.

The restricted predictability of TYMS inhibitor 5‐FU also suggests that training on a narrow selection of drugs with limited diversity in MoAs will inevitably struggle to accurately align new metabolic patterns unless the training data is expanded. Moreover, focusing on metabolomics analyzing only CCEM may further restrict the method's applicability. Nonetheless, many anticancer drugs currently in use either directly target CCEM or exhibit specific effects on downstream CCEM patterns, even when they act on non‐metabolic targets, as evidenced by MoAs such as AKT or Antimicrotubule. Thus, the present study aims to showcase the proof of concept of metabolomics and ML in preclinical drug discovery, considering the aforementioned limitations. Together, these results validate the approach, and its predictive power will further improve as the number of compounds and especially cell types used in the training sets increases.

Beyond ML‐based pattern recognition and MoA prediction, drug‐induced metabolic profiles contain important mechanistic information that may not be uncovered to this extent using high‐throughput methods without chromatographic separation. We illustrated this for different types of OXPHOS inhibition in relation to regulatory metabolic effects on cellular pyrimidine biosynthesis. For example, antimycin A was recently found to stimulate fumarate reduction by inhibiting CPLX III in 143B osteosarcoma cells, leading to re‐oxidation of ubiquinol to ubiquinone by CPLX II and SUC accumulation.^[^ ^58^ ^]^ In accordance with our study in PC‐3 cells, succinate (SUC) enrichment was also detected in CPLX III or fumarase mutant cells but not after CPLX I inhibition.^[^ ^59^ ^]^ Stable isotope labeling of these CPLX III‐compromised cells demonstrated that pyruvate carboxylase (EC 6.4.1.1) contributes to MAL and FUM formation, while SUC is produced from 2‐OG and from FUM, respectively, via clockwise and counterclockwise fluxes of the TCA cycle. Thus, the different patterns observed here for SUC, FUM, and MAL upon CPLX I compared with CPLX III inhibition can be attributed to the fact that fumarate reduction is possible only upon inhibition of CPLX III, while at the same time only a small fraction of FUM and MAL can be replenished by the reductive pathway under conditions of energy deficiency.^[^ ^59^ ^]^ Inhibition of succinate dehydrogenase (SDH, EC 1.3.5.1) in CPLX II leads to depletion of FUM and MAL and accumulation of SUC, as SDH is responsible for the conversion of succinate to fumarate. The activity of dihydroorotate dehydrogenase depends on the availability of CoQ as well as on its reduction state, i.e., the ratio of ubiquinone to ubiquinol (UQH2). This ratio was assessed in isolated mitochondria of 143B osteosarcoma cells, comparing partial SDH inhibition with pharmacological CPLX III inhibition. Remarkably elevated UQH2/CoQ ratios were exclusively observed with antimycin A‐mediated CPLX III inhibition but not with CPLX II inhibition alone.^[^ ^58^ ^]^ These findings align well with our observations, where CPLX III inhibition distinctly influences CA and DHO patterns compared with upstream CPLX I and CII inhibitions (Figure 3).

In conclusion, our methodology allows to correlate the metabolic impact of cytotoxic substances or even complex compound mixtures with pre‐defined MoAs, especially if these directly or indirectly affect the central carbon and energy metabolism. ML‐based pattern recognition not only enables the fine distinctiveness and prediction of MoAs, e.g. as shown by the differentiability of individual respiratory chain complexes. Mechanistic insights from metabolomics‐based MoA studies imply valuable opportunities for metabolic research, for example, to identify drugs for the development of combination chemotherapies. Therefore, our approach can significantly improve drug discovery and development by directing MoA studies toward the identification of specific molecular targets or pathways, and subsequent medicinal chemistry improvements of the lead compound, which is not restricted to cancer research.

## 4 Experimental Section

### Cell Culture

Cell handling and assay techniques were described in ref. [60]. The prostate cancer cell line PC‐3 (ATCC, Manassas, VA, USA), The estrogen receptor positivity (ER+) and HER2 negativity (HER2−) breast cancer cell line (ATCC, Manassas, VA, USA), and the ewing's sarcoma MHH‐ES1 (DSMZ, Braunschweig, Germany) were maintained in RPMI1640 basal cell culture medium supplemented with 10% FCS, 1% L‐glutamine and 1% penicillin/streptomycin. Cells were pre‐cultured in T‐75 cell culture flasks in a humidified atmosphere with 5% CO_2_ at 37 °C. The cells were routinely sub‐cultured when reaching 85% confluency with a maximum of six passages per batch. Adherent cells were washed with PBS and detached by trypsin/EDTA (0.05% in PBS).

### In Vitro Cell Viability Assays for IC_50_ Determination

Cells were seeded in 96‐well plates with a density of 6000 cells/100 µL medium per well and were left to attach overnight. Cells were then exposed to compounds of interest in a cell culture medium containing 0.5% DMSO using eight different concentration levels. These levels were compound‐specific and narrowed down to the range around IC_50_ specifically determined for each compound in preliminary cell viability assays. In parallel, cells were treated with 0.5% DMSO (negative control) and 100 µm digitonin (positive control, for data normalization, set equal to 0% cell viability). Each cell viability assay was performed with two biological replicates each with technical quadruplicates. After 48 h incubation time, cells were washed once with PBS before incubating with MTT (3‐(4,5‐dimethylthiazol‐2‐yl)‐2,5‐diphenyltetrazolium bromide) solution (0.5 mg mL^−1^) for 1 h at 37 °C and 5% CO_2_. Afterward, the MTT solution was discarded and DMSO was added to dissolve the formed formazan whose absorbance was measured at 570 nm in addition to a reference/background signal at 670 nm (SpectraMax M5, Molecular Devices, San Jose, CA, USA). For the crystal violet‐based cell viability assay (CV), the cells were fixed with 4% paraformaldehyde (PFA) for 20 min at RT after a single PBS wash. Then, they were dried for 10 min and stained with 10% crystal violet for another 20 min. The excess stain was removed by water wash and the cells were dried overnight at RT. The next day, acetic acid (33% v/v in aqua bidest.) was added to dissolve the stain, and the absorbance was measured at 570 and 670 nm as described before. The cell viability was calculated as a percentage in relation to untreated control cells. For data analyses and IC_50_ calculations, SigmaPlot 14.0 and Microsoft Excel 2013 were used. Mean values were calculated by using the four‐parametric logistic function.

### Cell Treatment for Metabolomics Experiment

Cells were seeded as hexuplicates in T‐25 cell culture flasks with a density of 0.5 million cells/5 mL. After cell attachment overnight, cells were treated with the compound of interest by applying it at IC_50_ final concentrations as obtained from earlier viability assays. Treatments and vehicle controls had the same final DMSO concentration (0.5%). After 48 h at 37 °C under 5% CO_2_, control flasks without test compound reached 85% confluence. Then the cell culture medium of the T‐25 flask was discarded and replaced by pre‐warmed PBS (37 °C) for a washing step that removed detached cells. Within a few seconds, the washing solution was discarded and the remaining cells were quenched with 1.5 ml cold acidic ethanolic solution (10% HCl of pH 1.4 in ethanol, −80 °C). The T‐25 flasks were sealed with watertight lids and then immersed in an ultrasonic water bath, with the flasks weighted with lead weights and centered in the ultrasonic field. Cells were quantitatively detached by 5 min ultra‐sonication (Bandelin Sonorex RK 106, 480 W, Berlin, Germany). Additionally, packed dry ice was placed in the water bath to maintain a low temperature close to 4 °C. Afterward, the suspension was transferred from each T‐25 flask into pre‐chilled 2 mL Eppendorf tubes kept on dry ice. These were subsequently placed in a sample concentrator at 4 °C (TurboVap LV, Biotage, Uppsala, Sweden), where the volume was reduced to 50 µL in an N_2_ stream. After volume reduction, two consecutive centrifugation steps were performed at 10600 rcf for 5 min at 1 °C, with another transfer of the supernatant to a fresh Eppendorf tube after the first centrifugation. Finally, the supernatant after the second centrifugation was transferred to an LC‐MS vial with inserts, and samples were frozen and stored at −80 °C until LC‐MS measurement.

### Metabolomics Assay

Separation of hydrophilic metabolites was performed by ion‐pairing chromatography on a Nucleoshell RP18 column (2.1 × 150 mm, particle size 2.1 µm, Macherey & Nagel, GmbH, Düren, Germany) using a Waters ACQUITY UPLC System, equipped with an ACQUITY Binary Solvent Manager and ACQUITY Sample Manager (5 µL injection volume; Waters GmbH, Eschborn, Germany). Eluents A and B were aqueous 10 mmol L^−1^ tributyl amine (adjusted to pH 6.2 with glacial acetic acid) and acetonitrile, respectively. Elution was performed isocratically for 2 min with 2% eluent B, from 2 to 18 min with a linear gradient up to 36% B, and from 18 to 21 min up to 95% B, and isocratically from 21 min to 22.5 min with 95% B, from 22.51 to 26 min again down to 2% B. The flow rate was set to 400 µL min^−1^, and the column temperature was maintained at 40 °C.

Mass spectrometric analyses of small molecules were performed by targeted MS/MS via multiple reaction monitoring (MRM) by using a QTRAP 6500 (AB Sciex GmbH, Darmstadt, Germany) operating in negative ionization mode and controlled by Analyst 1.7.1 (AB Sciex GmbH, Darmstadt, Germany) (Table S2, Supporting Information). The source operation parameters were the following: ion spray voltage, −4500 V; nebulizing gas, 60 psi; source temperature, 450 °C; drying gas, 70 psi; curtain gas, 35 psi.

Peak integration was performed using the MultiQuant software version 3.0.3 (Sciex, Toronto, CA). To account for different cell numbers in treatments and vehicle controls, individual CCEM peak areas were normalized to the total peak area for each sample. Finally, each normalized metabolite area in an individual sample was divided by the mean of the likewise normalized signal area of all vehicle control samples of the respective experimental set. All area ratio data were logarithmized to the basis of 2. The statistical analysis and graphical presentation were performed by MetaboAnalyst 5.0 using the log2‐normalized data and Range Scaling.^[^ ^61^ ^]^

### Lipidomics Assay

PC‐3 cells were quenched with 1.5 mL cold MeOH (−80 °C) and dispersed by ultrasonication as described above. Cell debris and solution were then transferred together into a new tube and were dried in a nitrogen stream. After the addition of one steel bead (3 mm), three steel beads (1 mm), and 200 mg of glass beads (0.75–1 mm), 700 µL MTBE and 200 uL of water were added, and cryo bead milling for 3 × 20 s was performed (MP24, Biomedicals Inc., 4.0 m s^−1^). Following phase separation by centrifugation (2 min, 10000 x g) the upper phase was collected and stored on ice. The lower phase was re‐extracted with another 700 µL MTBE and, after centrifugation, the organic layers were combined and dried in a nitrogen stream. For analysis, the dry residue was dissolved in 500 µL MeCN/2‐propanol/water (60/35/5).

Lipid separation on UPLC was performed according to ref. [62] with the following modifications. Eluent A consisted of 60% MeCN and 40% water, and eluent B consisted of 90% 2‐propanol and 10% MeCN, both with 10 mm ammonium formate and 0.1% formic acid. Separation was carried out on a Nucleoshell RP18 (Macherey & Nagel, Dueren, Germany) with the dimensions 2 mm × 150 mm × 2.7 µm. The gradient on a Waters Acquity was: 0–1.5 min 32% B, 1.5–18 min linear gradient to 98% B, 18–20 min 98% B, 20–24 min 32% B with 10 µL injection volume and 40 °C oven temperature.

Separated lipids were ionized by electrospray ionization and analyzed in positive ionization mode by HR QToF‐MS/MS (Zeno7600, Sciex, Toronto) using these source parameters: ion source gas 1, 60 psi; ion source gas 2, 70 psi; curtain gas, 35 units; CAD gas, 7 units; temperature 450 °C, ion spray voltage floating, −5500 V. MS1 and CID‐MS/MS spectra were scanned from 65–1500 Da using 100 ms accumulation time for MS1 and 20 ms each for a maximum of 40 data‐dependent MS/MS experiments. For MS1 survey scans and MS/MS the declustering potential (DP) was 80 V with a spread of 50 V, whereas in MS1 the collision energy was fixed to 10 V. MS/MS scans were acquired with a collision energy of 35 V, and a spread of 25 V. Mass accuracy was recalibrated after every five samples using internal standards provided for ESI by Sciex. For lipidomics data analysis, raw data were processed by MS‐Dial version 4.9 using the lipidomics standard library embedded in the program.^[^ ^63^ ^]^ Peak height tables with classified lipids were exported from MS‐Dial, normalized to cell number, and processed in GraphPad Prism 9.1.5.

### ^13^C Labeling

PC‐3 cells were seeded in T‐25 cell culture flasks as described above. After 24 h of incubation, cell culture medium was exchanged against RPMI 1640 medium without glucose (Gibco, Darmstadt, Germany), supplemented with 10% heat‐inactivated dialyzed FBS (Gibco, Darmstadt, Germany) and uniformly labeled D‐glucose (U‐^13^C6, 99%) (Cambridge Isotope Laboratories, Inc., Massachusetts, USA) at a final concentration of 2 g L^−1^. Simultaneously, samples were treated with BETA at the IC_50_ concentration (Table S1, Supporting Information), whereas in the control flasks no BETA was added. Isotopolog analysis was performed using Lipidomics extraction 48 h post‐treatment, following the protocol described above. The samples were analyzed using UPLC‐ToF in positive mode ESI with the chromatographic gradient as described. Isotopologs of the three most abundant PC (m/z;r.t.: 760.5836; 12.65 min, m/z;r.t.: 788.6158; 13.59 min, and m/z;r.t.: 732.5521; 11.78 min) were area integrated based on their theoretically expected [M+H] ions within a mass tolerance of 10 ppm. The data analysis was done using Sciex OS. The percentual ^13^C incorporation was calculated as described in^[^ ^64^ ^]^ and visualized by GraphPad Prism 10.

### CoQ Assay

Coenzyme Q10 was analyzed by UPLC‐QToF with positive mode ESI. The gradient was the same as described in the lipidomics section. CoQ was determined by [M+H] of 863.692 with a mass tolerance of 2 ppm. The retention time and the MS/MS spectrum were compared to an authentic standard (Merck). Peak area values of pentuplicate treatments were normalized to the cell number.

### Cell Counts

PC‐3 cells of three parallel samples in each control/treatment group were detached by trypsination for 3 min in 0.05% trypsin/EDTA and 37 °C. After trypsination stop with FCS‐containing culture medium, the resuspended cells were stained with Trypan blue, and cell counting was performed using a Neubauer counting chamber (Marienfeld, Lauda‐Königshofen, Germany).

### Localization of AAHR in PC‐3 Cells

PC‐3 cells were seeded in a 10‐well chamber slide with 1000 cells per well and allowed to grow at 37 °C and 5% CO_2_ for 24 h. Cells were treated with 0.1 µm AAHR for 4 h followed by washing twice with PBS. Staining with 0.1 µm MitoTracker Green FM was done for 15 min. After washing with PBS twice, fresh medium was added. Fluorescence was recorded using an LSM900 (Carl Zeiss, Jena, Germany) with ex/em 561 nm/576–700 nm for AAHR and ex/em 488 nm/490–540 nm for MitoTracker Green. Co‐localization of AAHR and MitoTracker Green was analyzed using Zen Blue image analysis software (Zeiss).

### Seahorse Analysis

To investigate the compound‐induced alterations in cellular energy metabolism, the Seahorse cell analysis technology platform from Agilent was utilized, applying the Seahorse XF Cell Mito Stress Test Kit (Agilent Technologies, Santa Clara, CA, USA). The analyses were performed on a Seahorse XF 96 – Extracellular Flux Analyzer according to instructions of the manufacturer. PC‐3 cells were seeded in specific 96‐well plates at day 0 using a density of 12000 cells per well. Subsequently, starting at day 1, the cells were treated with 20 nm and 100 nm of AAHR for 24 h (a duration not yet causing apparent signs of cytotoxicity), or were left untreated, and finally were measured at day 2. Another sample of untreated cells was used to assess mitochondrial function after acute compound treatment. For this purpose, one of the standard chemicals was replaced by AAHR, respectively.

The Seahorse XF Cell Mito Stress Test was performed, which was based on the measurement of oxygen consumption rate (OCR) and extracellular acidification rate (ECAR) of viable cells during sequential manipulation with specific chemicals to obtain information about mitochondrial function and to quantify cellular bioenergy levels (ATP).

To further explore the precise mechanism of inhibition of mitochondrial respiration, the Mito Stress Test was modified to analyze the impact of acute AAHR treatment on OCR and ECAR. The standard assay comprises a consecutive treatment with oligomycin (complex V / ATP synthase inhibitor), FCCP (uncoupling agent), and rotenone/antimycin A (complex I / III inhibitors) leading to a reduction of OCR (increase of ECAR), strong increase of OCR to a maximum, and complete decrease of OCR to a minimum, respectively.

### Docking Studies

Molecular docking was performed in YASARA software^[^ ^65^ ^]^ version 20.12.24 by using the AutoDockLGA algorithm,^[^ ^66^ ^]^ and AMBER14 force field.^[^ ^67^ ^]^ The cryo‐EM structure of human CEPT1 complexed with CDP‐choline (PDB ID: 8GYW),^[^ ^68^ ^]^ and the AlphaFold^[^ ^69^ ^]^ model for CPT1 which was obtained from UniProt (accession number Q8WUD6) were utilized for molecular docking studies. The AlphaFill^[^ ^70^ ^]^ server was used to assign the Mg^2+^ ion in CPT1, while it (Mg^2+^) exists in the CEPT1 cryo‐EM structure. The binding site was defined according to the location of the ligand (CDP‐choline) in the cryo‐EM structure of CEPT1. Ligands and protein structures were energy‐minimized, and a simulation box (10 Å) was defined around the Mg^2+^ ion. The validation of the docking protocol was performed by re‐docking the ligand in the cryo‐EM (CDP‐choline) of CEPT1 (Figure S16, Supporting Information), using the same parameters. The results from 100 runs were clustered using an RMSD (Root Mean Square Deviation) of 2.5 Å, and MOE (Molecular Operating Environment) 2020 software^[^ ^71^ ^]^ was employed to investigate the interaction between the ligands and the proteins.

### Classification Methods and Prediction Analysis—Normalization of CCEM Profiles

CCEM profiles were normalized per metabolite by subtracting the mean across all drugs/replicates and dividing the result by the range between the maximum and minimum values across all replicates, i.e.,

(1) $$n_{m,r}=\frac{x_{m,r}-{\bar{x}}_{m}}{\underset{s}{max}x_{m,s}-\underset{s}{min}x_{m,s}}$$

where *x*_*m*,*r*_ denotes the input value for metabolite *m* in experiment *r* and ${\bar{x}}_{m}=\frac{1}{R}\;\sum_{r\;=\;1}^{R}x_{m,r}$

The same coefficients were also used for normalizing the data in the prediction set to avoid re‐scaling and subsequent re‐training of models when processing prediction sets. For predictions in other cell models, a cell line‐specific mean value was determined to account for different base profiles.

### Classification Methods and Prediction Analysis—Correlation Analysis of Metabolites

For all pairs of metabolites, Pearson correlation was computed between their profiles across all drugs/replicates. Visualization as a heatmap and corresponding clustering of rows/columns was performed using the *pheatmap* R package (v. 1.0.12).^[^ ^72^ ^]^

### Classification Methods and Prediction Analysis—Hierarchical Clustering of CCEM Profiles

Normalized CCEM profiles of all drugs/replicates were clustered using Euclidean distance and the *hclust* R function with method “ward.D”. The resulting cluster trees were visualized as dendrograms. Here, leaves are annotated with the MoA of the corresponding drug. Inner nodes of the cluster tree, under which all leaves belong to the same MoA are annotated accordingly. To generate a more condensed view on the clustering result, each sub‐tree in which all leaves belong to the same drug was aggregated into a single leaf. For all drugs, this results in a single leaf representing all replicates of that drug, except for PRFN/ORID. The latter has CCEM profiles so similar that replicates of both drugs appear intermixed. Heatmaps of selected profiles were generated using MetaboAnalyst 5.0. Peak integrated raw data were normalized to the sum of all signals as to compensate for the different cell numbers. Log_2_‐fold changes between six replicate treatments per drug and six controls, both sampled after 48 h, were rank‐scaled (Table S3, Supporting Information).

### Random Forest – Training

Random Forests were trained using the *randomForest* function of the corresponding R package (v. 4.7–1.1)^[^ ^73^ ^]^ using 1000 trees balancing between runtime and stability. Input data of the Random Forest method were the CCEM profiles of all replicates of all drugs in the training set. True class labels during training were the corresponding drugs, i.e., the Random Forest was trained to distinguish individual drugs, which in turn had been assigned to a specific MoA.

### Random Forest – Prediction

Each normalized CCEM profile of each replicate for each drug in the prediction set was considered independently, and probabilities for individual drugs were predicted using the trained Random Forest model using the *predict* method with parameter “type = ’prob’”. Returned probabilities were averaged across all replicate CCEM profiles of each drug, and then averaged across all drugs with a common MoA in the training set to yield the probability of a specific MoA (Table S3, Supporting Information). The two‐level averaging ensures that all drugs have the same influence on the prediction result, irrespective of the number of replicate measurements.

### Lasso‐Based Approach – Training & Prediction

For the Lasso‐based approach, the regression coefficients themselves were used to predict the MoA of a new drug. Here, the CCEM profile of an individual replicate of the drug in the prediction set was considered as the response variable, and the CCEM profiles of all replicates of all drugs in the training set were considered as the input matrix of a Lasso regression using the glmnet function from the corresponding R package (v. 4.1–4).^[^ ^74^ ^]^ Regression coefficients were limited to non‐negative values by setting the parameter for the lower boundary of regression coefficients to zero. The regularization parameter was set to a fixed value within the range of auto‐computed values (λ = 0.025) and no intercept value was considered by setting “intercept = FALSE”. Regression coefficients were obtained using the *coef* method. Returned coefficients were averaged across all replicate CCEM profiles of each drug, and then averaged across all drugs with a common MoA to yield the prediction score of a specific MoA. For visualization purposes, prediction scores were normalized to a sum of one across all MoAs since each drug is represented by 5–6 replicates in the training set.

### k‐Nearest Neighbor – Training & Prediction

Predictions of the k‐nearest neighbor classifier were obtained from the function *knn* of the *class* R package^[^ ^75^ ^]^ using the CCEM profiles of each replicate of each drug in the training set and corresponding MoA as reference. The number of nearest neighbors was set to “k = 5” to account for the number of replicates per drug.^[^ ^74^ ^]^

### Leave‐One‐Out Cross Validation

For evaluating the prediction performance of each approach on the training set, a leave‐one‐out cross‐validation over the drugs in the training set was performed. To avoid data leakage from the training partition to the test partition within individual iterations of the cross‐validation, all replicates of a drug were excluded from the training partition and used as the test partition in a leave‐one‐out cross‐validation. Specifically, all replicate CCEM profiles of one drug with known MoA were considered as prediction partitions, and the replicate CCEM profiles of all remaining drugs were used as the corresponding training partition. For each replicate of the drug in the prediction partition, predictions were obtained from the three alternative methods and those were compared against its true MoA. R source code of the evaluation procedure is provided as Data S1 (Supporting Information).

## Conflict of Interest

The authors declare no conflict of interest.

## Author Contributions

M.S., G.U.B., L.A.W., A.T., and R.R. conceptualized the project. M.S. performed cell cultures, metabolomics assays and data analyses. J.G. and I.G. performed the machine learning. T.M. performed the Seahorse assays. M.S. and B.H. performed the LMS imaging. M.Y. and M.D. performed the docking studies. M.S. and G.B. performed and analyzed the lipidomics assays. R.C. and L.R. provided AAHR and Breastin, respectively. M.S., G.U.B. and J.G. wrote the original draft. G.U.B.; L.A.W. and R.R. supervised the work, designed the experiments, reviewed and edited the draft. L.A.W., A.T., I.G. and R.R. reviewed the manuscript. L.A.W. and A.T. acquired the funding and administered the project. All authors have read and agreed to the published version of the manuscript.

## Supporting information



Supporting Information

Supplemental Table 1

Supplemental Table 2

Supplemental Table 3

Supplemental Table 4

Supplemental Table 5

Supplemental Table 6

## Data Availability

The data that support the findings of this study are available in the supplementary material of this article.
