# Supplementary material for: Advancing Anticancer Drug Discovery: Leveraging Metabolomics and Machine Learning for Mode of Action Prediction by Pattern Recognition
Source: Adv Sci (Weinh). 2024 Oct 21;11(47):2404085. doi: 10.1002/advs.202404085 (PMC11653622; doi:10.1002/advs.202404085)
Supplement: Supplementary file 1 — Supporting Information [file ADVS-11-2404085-s005.pdf]

ADVANCED SCIENCE
Open Access

Supporting Information

for *Adv. Sci.*, DOI 10.1002/advs.202404085

Advancing Anticancer Drug Discovery: Leveraging Metabolomics and Machine Learning for Mode of Action Prediction by Pattern Recognition

Mohamad Saoud, Jan Grau, Robert Rennert, Thomas Mueller, Mohammad Yousefi, Mehdi D. Davari, Bettina Hause, René Csuk, Luay Rashan, Ivo Grosse, Alain Tissier, Ludger A. Wessjohann and Gerd U. Balcke**

Supporting Information for

Advancing Anticancer Drug Discovery: Leveraging Metabolomics and Machine Learning for Mode of Action Prediction by Pattern Recognition

Mohamad Saoud[1], Jan Grau[3], Robert Rennert[1], Thomas Mueller[4], Mohammad Yousefi[1], Mehdi D. Davari[1], Bettina Hause[2], René Csuk[5], Luay Rashan[6], Ivo Grosse[3], Alain Tissier[2], Ludger A. Wessjohann[1*] and Gerd U. Balcke[2*]

[1,2] Leibniz Institute of Plant Biochemistry, Dept. of Bioorganic Chemistry[1], Dept. of Cell and Metabolic Biology[2], Weinberg 3, 06120 Halle (Saale), Germany

[3] Martin Luther University Halle-Wittenberg, Institute of Computer Science, 06120 Halle (Saale), Germany

[4] Martin Luther University Halle-Wittenberg, Medical Faculty, University Clinic for Internal Medicine IV (Hematology/Oncology), 06120 Halle (Saale), Germany

[5] Martin Luther University Halle-Wittenberg, Institute of Chemistry, Department of Organic and Bioorganic Chemistry, 06120 Halle (Saale), Germany.

[6] Dhofar University, Research Center, Frankincense Biodiversity Unit, Salalah 211, Oman

*Ludger A. Wessjohann, Gerd U. Balcke,
Leibniz Institute of Plant Biochemistry, Weinberg 3, D-06120 Halle/S., Tel.: +49(0)345 5582 1510
Email: gerd.balcke@ipb-halle.de,

This PDF file includes:

Figures S1 to S15

Other additional files for this manuscript include the following:

Tables S1 to S5

Table S1 (separate file). IC_{50} values, MeA and structural information for training and prediction compounds.

Table S2 (separate file). Structural information and LC-MS/MS parameters of analyzed CCEM metabolites.

Table S3 (separate file). Sum-normalized raw data (peak area) of training and prediction compounds.

Table S4 (separate file). Binding affinity of triterpenoid ligands to the CEPT1 and CPT1 enzymes, in comparison to the native substrate CDP-choline.

Table S5 (separate file). Cell number-normalized lipidomics data of control, BA and AAHR treated PC-3.

Supporting Information

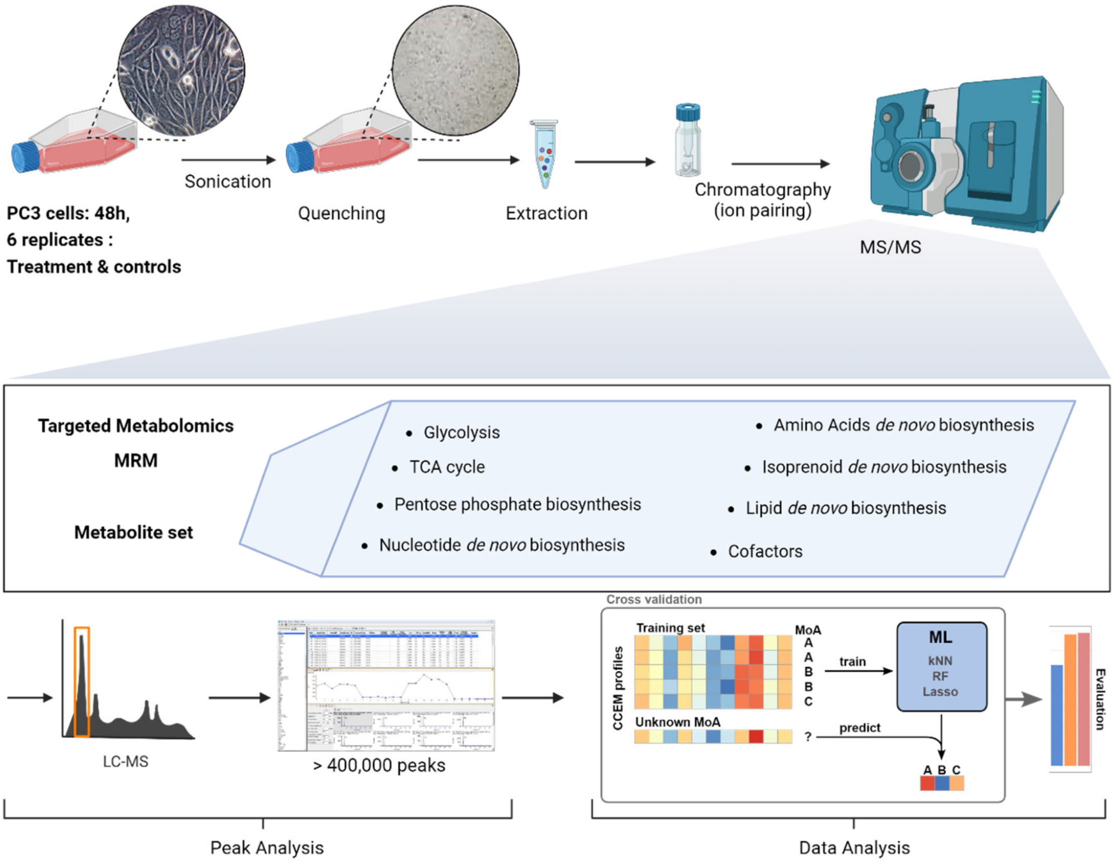

Fig. S1A. Analysis workflow for MoA prediction. This graph was created with BioRender.com.

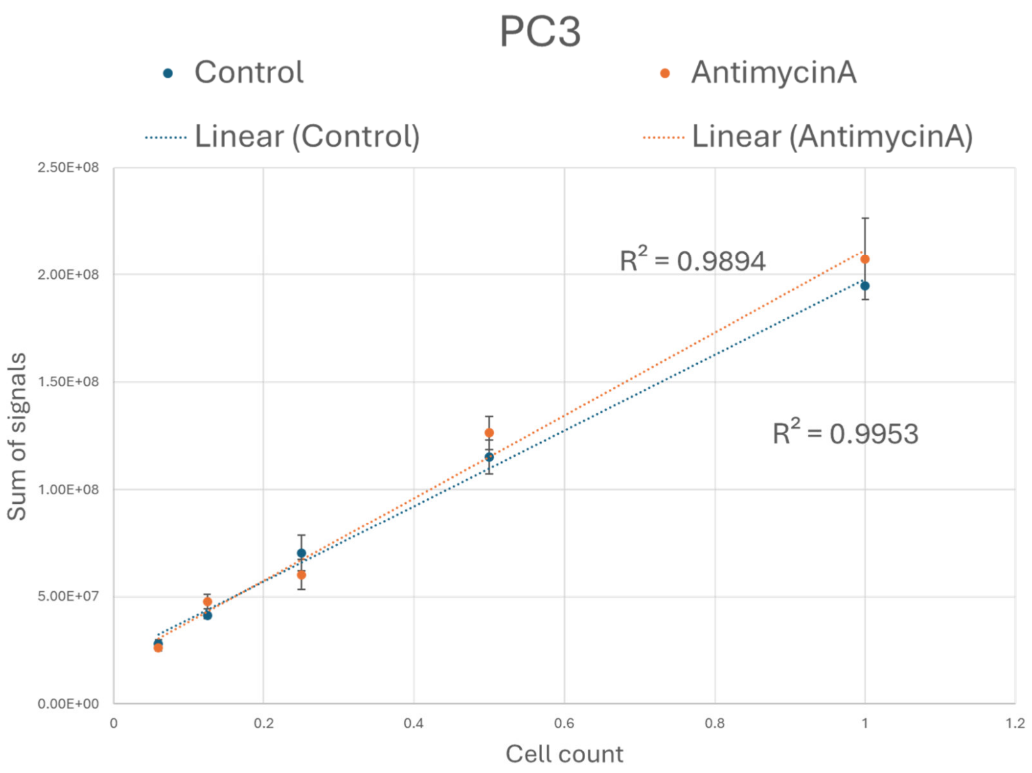

Fig. S1B. Validity of Normalization Method illustrating the sum of all integrated metabolite peak areas correlates with cell number independent of the treatment.

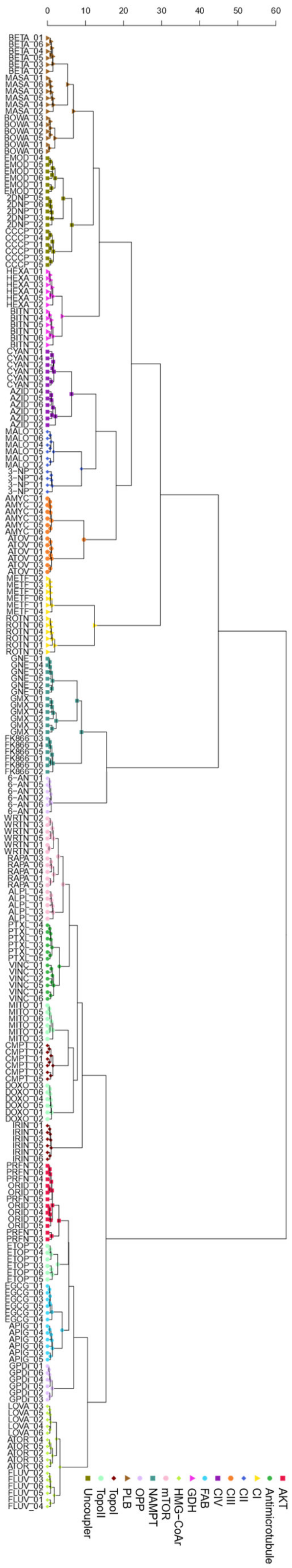

Fig. S2. Hierarchical cluster analysis of metabolic patterns of 38 reference compounds inhibiting different molecular targets of cancer metabolism in prostate cancer cells (PC-3) including all individual sample data.

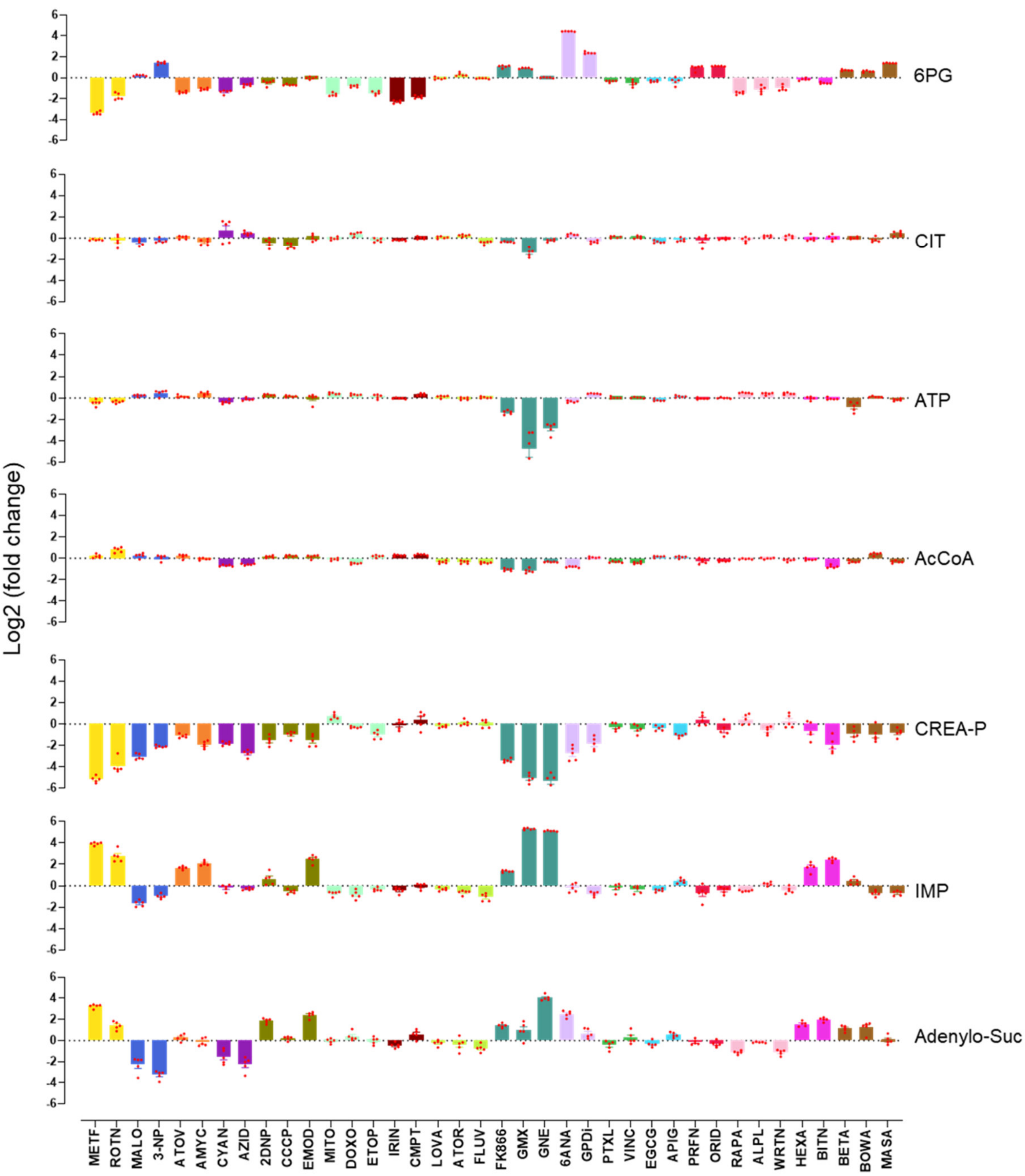

Fig. S3. Relative abundance of selected metabolites across all training data. Bars represent the average log_2-fold change of normalized peak areas of n=6 treatments and controls after 48 h incubation.

Compounds: BETA – betulinic acid, MASA – maslinic acid, BOWA – boswellic acid, EMOD – emodin, 2DNP – 2,4-dinitrophenol, CCCP – carbonyl cyanide chlorophenylhydrazone, HEXA – hexachlorophene, BITN - bithionol, CYAN – potassium cyanide, AZID – sodium azide, MALO – malonic acid, 3-NP – 3-nitropropionic acid, AMYC – antimycin A, ATOV – atovaquone , METF – metformin, ROTN – rotenone, GNE – GNE-617, GMX – GMX1778, FK866 – FK866, 6ANA – 6-aminonicotinamide, WRTN – wortmannin, RAPA – rapamycin, ALPL – alpelisib, PTXL – paclitaxel, VINC – vincristin, MITO – mitoxantrone, CMPT – camptothecin, DOXO – doxorubicin,

IRIN – irinotecan, PRFN (PR) – perifosine, ORID (OR) – oridonin, ETOP – etoposid, EGCG – epigallocatechin gallate, APIG – apigenin, GPDi – glucose-6-phosphate dehydrogenase inhibitor, LOVA – lovastatin, ATOR – atorvastatin, FLUV – fluvastatin.
MoA: AKT – protein kinase B (AKT), Antimicrotubule, CPLX I – complex I, CPLX II – complex II, CPLX III – complex III, CPLX IV – complex IV, FAB – fatty acid biosynthesis, GDH – glutamate dehydrogenase, HMG-CoAr – HMG-CoA reductase, mTOR – PI3K/mTOR signaling, NAMPT – nicotinamide phosphoribosyltransferase, OPP – oxidative pentose phosphate pathway, PLB – phospholipid biosynthesis, TopoI – topoisomerase I, TopoII – topoisomerase II, uncoupler – uncoupling of oxidative phosphorylation.

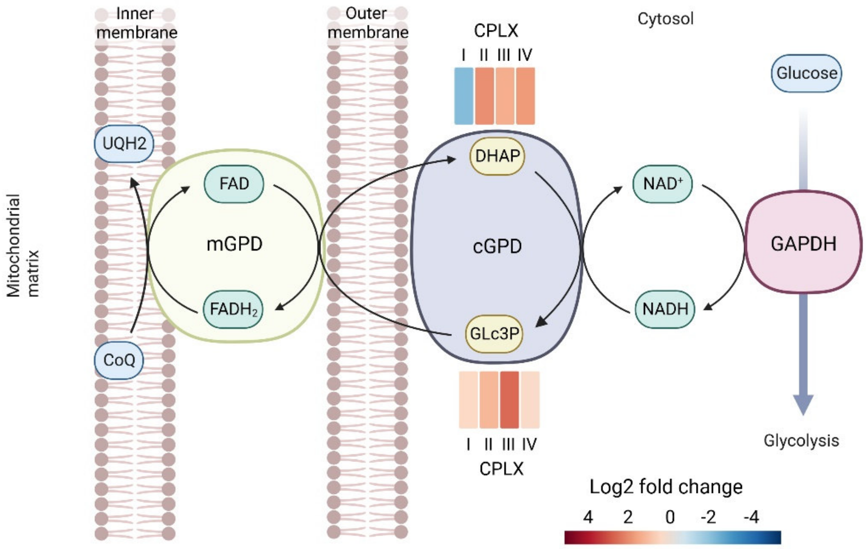

Fig. S4. The glycerol phosphate shuttle antagonizes mitochondrial dysfunction in cancer cells (31). By using cytosolic NADH, e.g., as a side-product of glycolysis, to reduce DHAP to glycerol 3-phosphate (Glc3P) cytosolic NAD is regenerated, which supports ongoing glycolytic flow. Glc3P can pass electrons to mitochondrial glycerol 3-phosphate dehydrogenase (EC 1.1.5.3), transferring reducing power to $FADH_2$. $FADH_2$ finally reduces CoQ at the inner mitochondrial membrane, which also regenerates DHAP. This graph was created with BioRender.com.

A

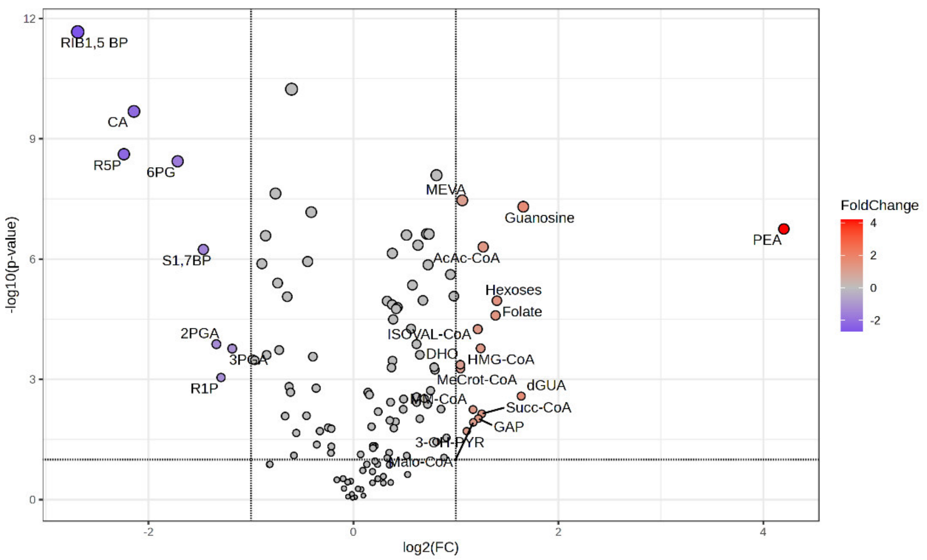

RIB1,5 BP
CA
R5P
6PG
S1,7BP
2PGA
3PGA
R1P
MEVA
Guanosine
AcAc-CoA
Hexoses
Folate
ISOVAL-CoA
DHO
HMG-CoA
MeCrot-CoA
dGUA
Succ-CoA
GAP
3-OH-PYR
Malo-CoA
PEA
-log10(p-value)
log2(FC)
FoldChange

B

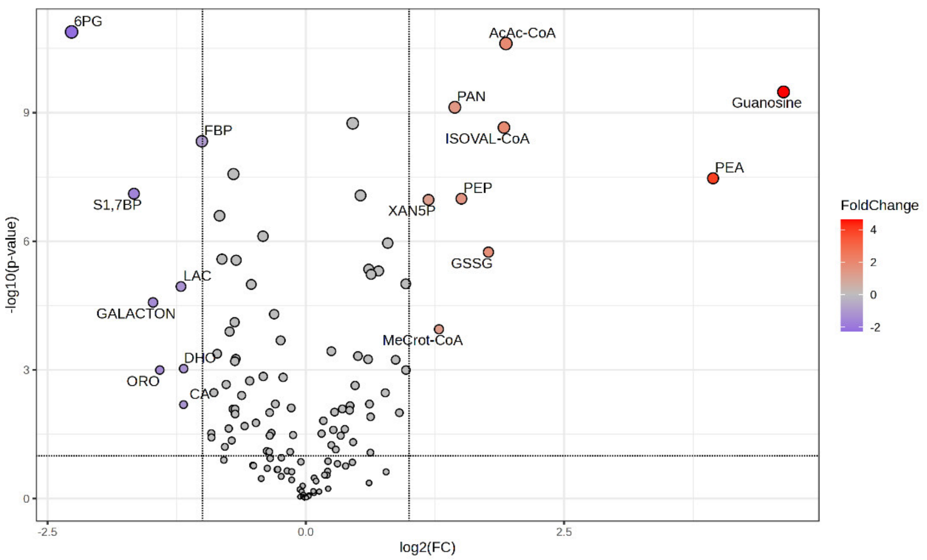

6PG
AcAc-CoA
PAN
Guanosine
FBP
ISOVAL-CoA
PEA
S1,7BP
PEP
XAN5P
GSSG
LAC
GALACTON
MeCrot-CoA
DHO
ORO
CA
-log10(p-value)
log2(FC)
FoldChange

C

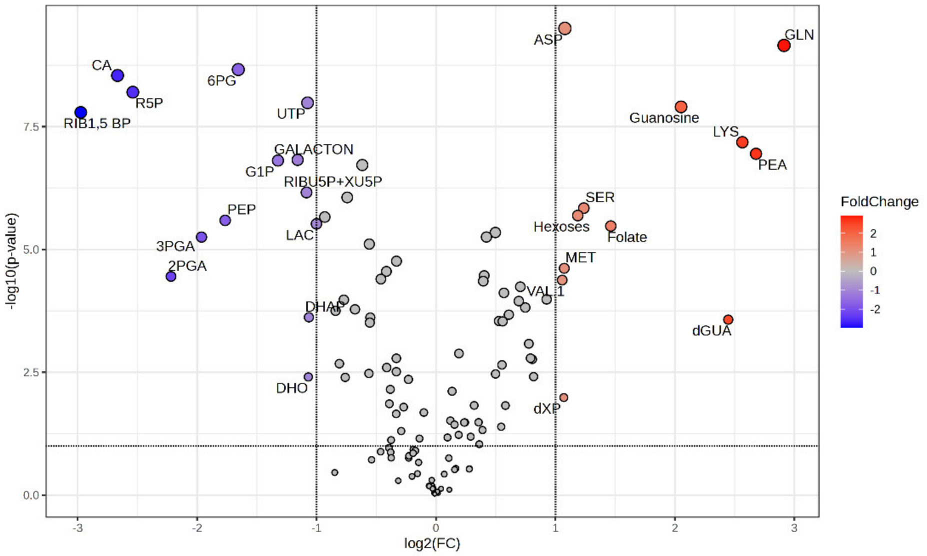
CA
6PG
R5P
UTP
RIB1,5 BP
GALACTON
G1P
RIBU5P+XU5P
PEP
LAC
3PGA
2PGA
DHAP
DHO
ASP
GLN
Guanosine
LYS
PEA
SER
Hexoses
Folate
MET
VAL 1
dGUA
dXP
-log10(p-value)
log2(FC)
FoldChange
2
1
0
-1
-2

D

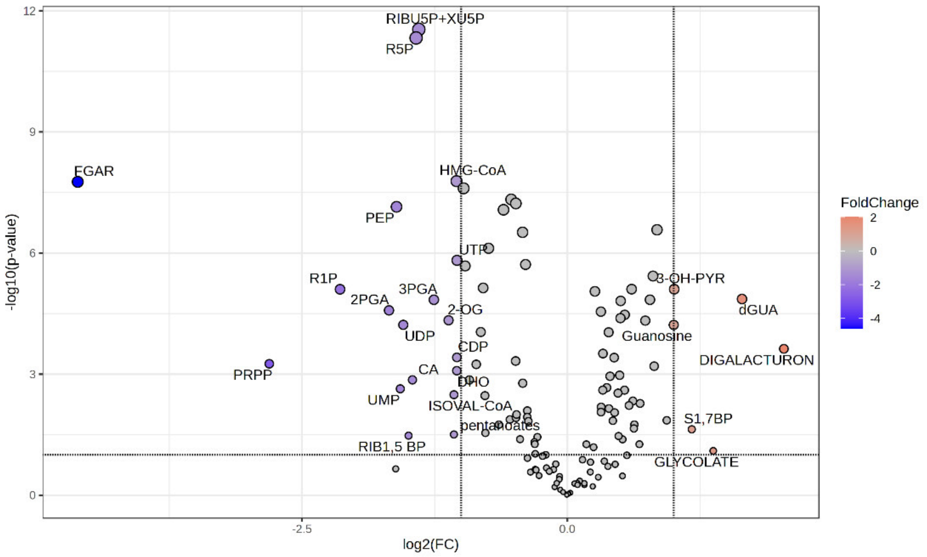
RIBU5P+XU5P
R5P
FGAR
HMG-CoA
PEP
UTP
R1P
3PGA
2PGA
2-OG
UDP
CDP
PRPP
CA
DHO
UMP
ISOVAL-CoA
pentanoates
RIB1,5 BP
3-OH-PYR
dGUA
Guanosine
DIGALACTURON
S1,7BP
GLYCOLATE
-log10(p-value)
log2(FC)
FoldChange
2
0
-2
-4

E

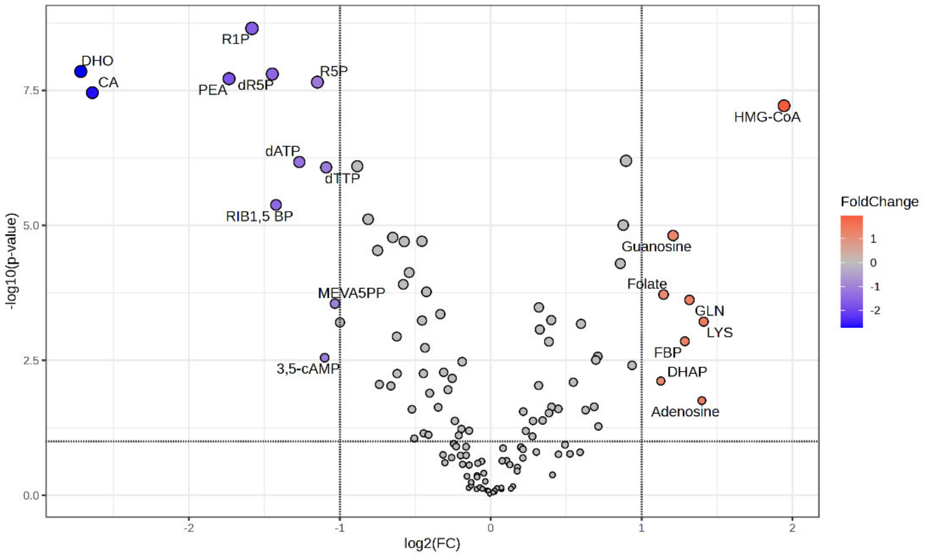
R1P
DHO
CA
PEA
dR5P
R5P
HMG-CoA
dATP
dTTP
RIB1,5 BP
Guanosine
Folate
MEVA5PP
GLN
LYS
FBP
3,5-cAMP
DHAP
Adenosine
-log10(p-value)
log2(FC)
FoldChange

F

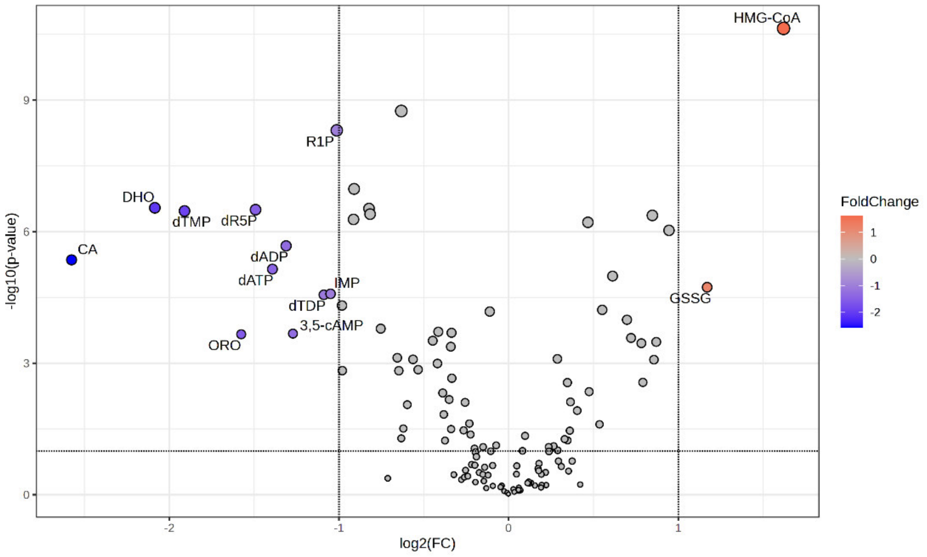
HMG-CoA
R1P
DHO
dTMP
dR5P
CA
dADP
dATP
IMP
GSSG
dTDP
3,5-cAMP
ORO
-log10(p-value)
log2(FC)
FoldChange

G

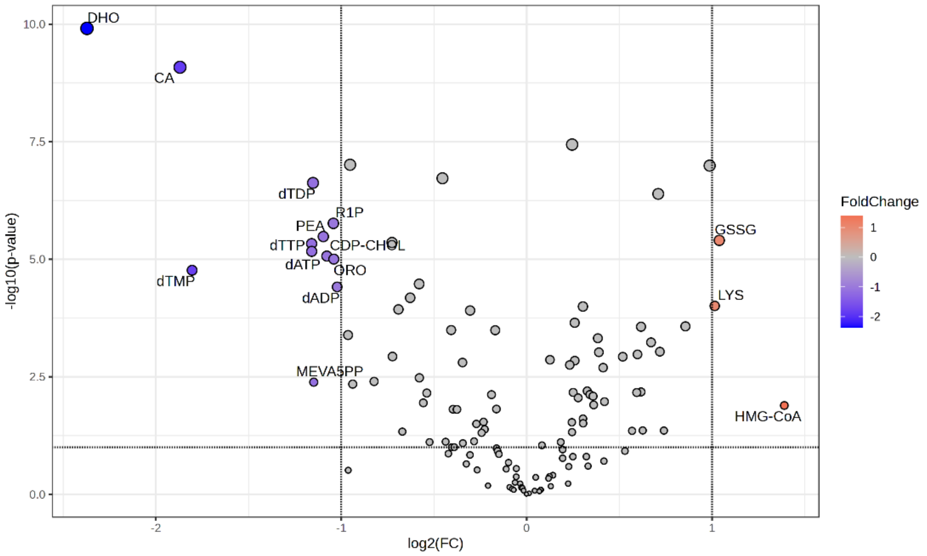
DHO
CA
dTDP
R1P
PEA
dTTP
CDP-CHOL
dATP
ORO
dTMP
dADP
GSSG
LYS
MEVA5PP
HMG-CoA
FoldChange
-log10(p-value)
log2(FC)

H

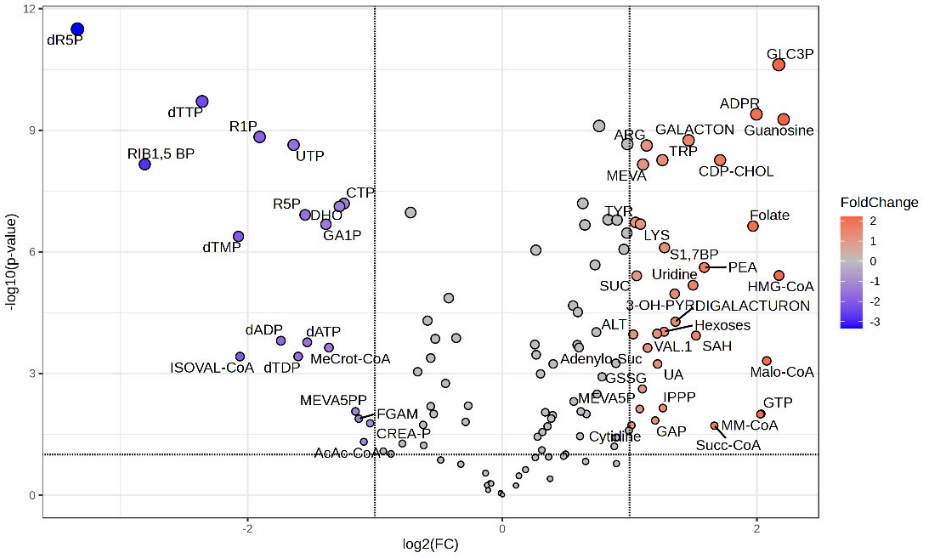
dR5P
GLC3P
dTTP
ADPR
R1P
GALACTON
Guanosine
ARG
RIB1,5 BP
UTP
TRP
MEVA
CDP-CHOL
CTP
R5P
DHO
TYR
Folate
GA1P
LYS
dTMP
S1,7BP
PEA
Uridine
SUC
HMG-CoA
3-OH-PYR
DIGALACTURON
ALT
Hexoses
dADP
dATP
VAL.1
SAH
MeCrot-CoA
Adenylo-Suc
ISOVAL-CoA
dTDP
GSSG
UA
Malo-CoA
MEVA5PP
MEVA5P
IPPP
GTP
FGAM
MM-CoA
CREA-P
GAP
Cytidine
Succ-CoA
AcAc-CoA
FoldChange
-log10(p-value)
log2(FC)

I

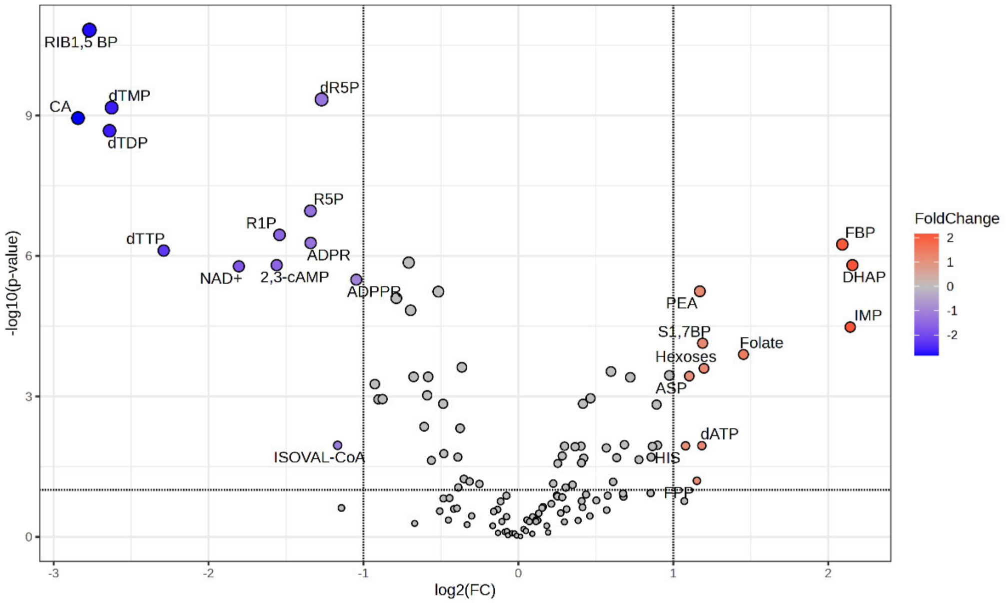

Fig. S5. Volcano plots representing relative metabolite abundances (average of n = 6 replicates) after 48 h in selected treatments versus non-treated vehicle controls, represented with accumulation (red), depletion (blue) and non-significant changes (gray) following the 48 h treatment with

Topoisomerase I inhibitors: **(A)** camptothecin, **(B)** irinotecan,

Topoisomerase II inhibitors: **(C)** mitoxantrone, **(D)** doxorubicin,

HMG-CoA reductase inhibitors: **(E)** lovastatin (LOVA), **(F)** fluvastatin (FLUV), **(G)** atorvastatin (ATOR),

and putative PLB inhibitors **(H)** betulinic acid (BETA),

TYMS inhibitor **(I)** 5-fluorouracil (5-FU).

A legend for the metabolites' abbreviations is given in Table S2.

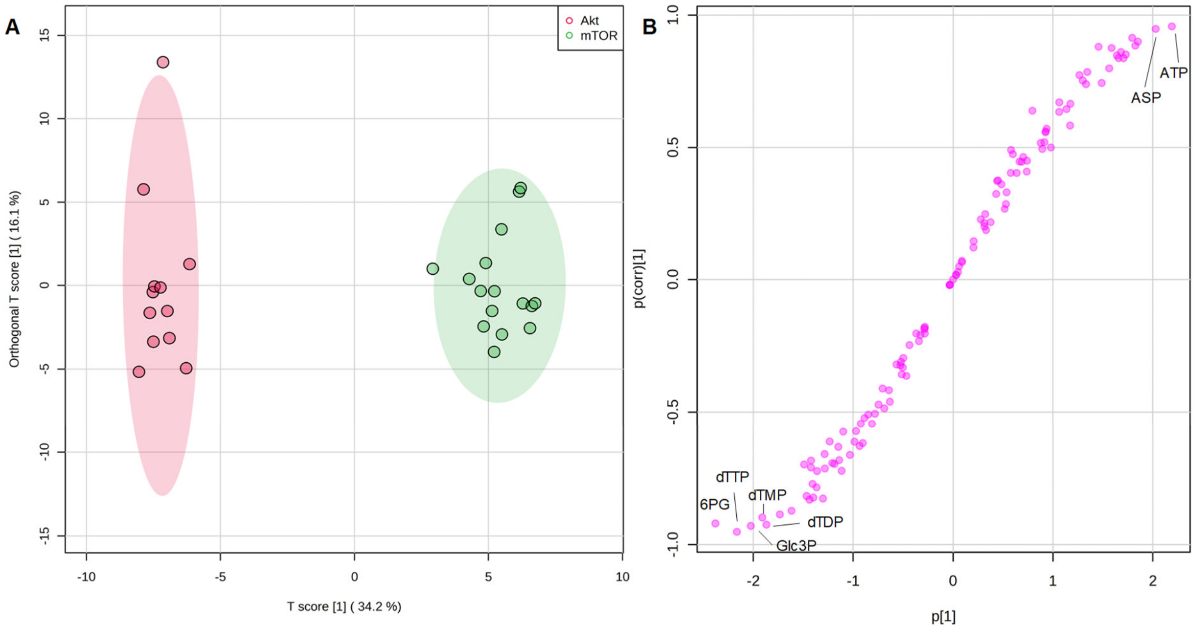

Fig. S6. Orthogonal partial least squares (oPLS-DA) discrimination between all AKT inhibitors and all mTOR inhibitors, **(A)** scores, **(B)** loadings (importance of variables). For the analysis, all 48 h data of wortmannin, rapamycin and alpelisip were grouped as mTOR and compared against all data of perfosine and oridonin, grouped as AKT. Log2-fold changes vs. non-treated controls were range-scaled.

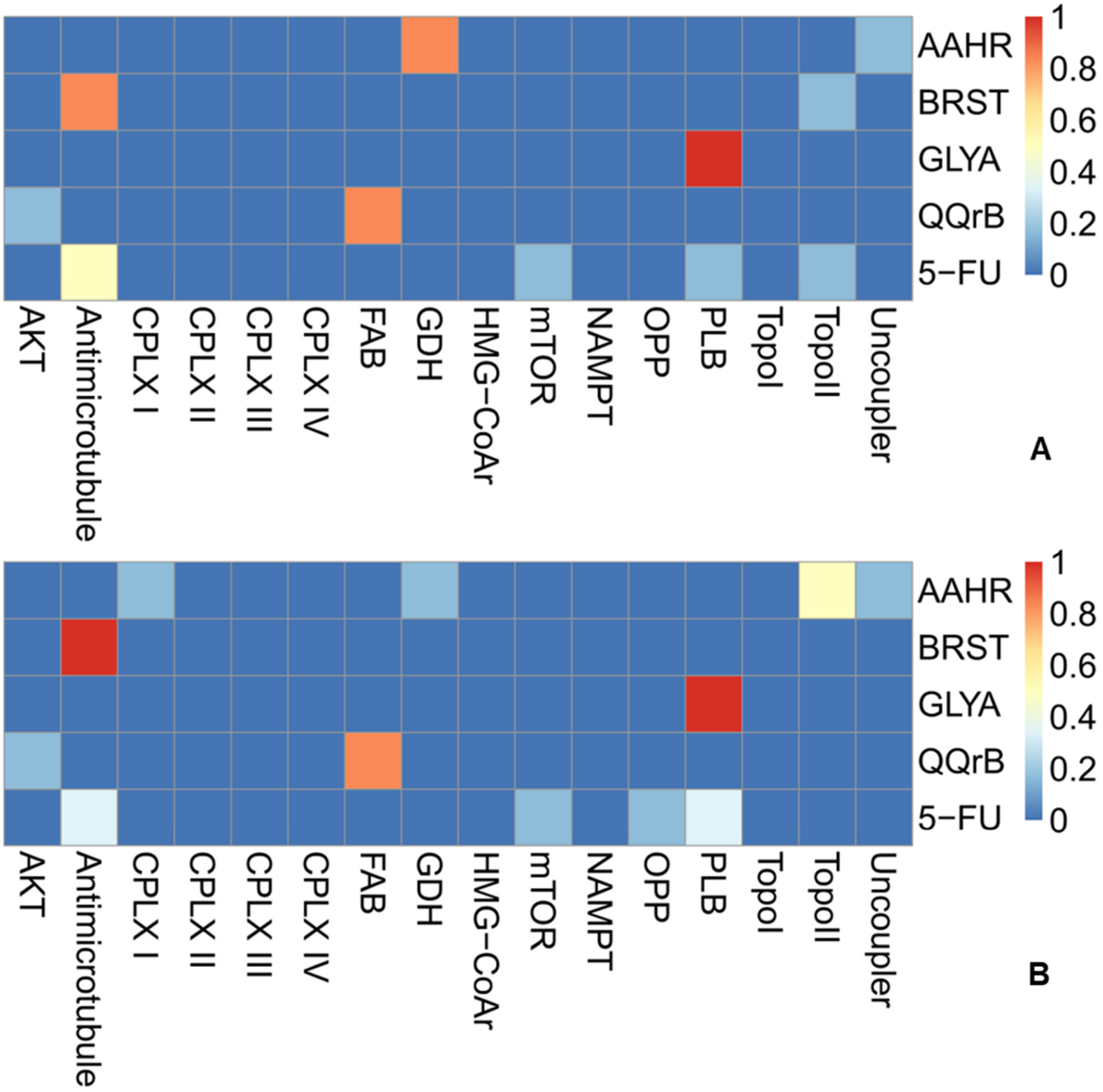

Fig. S7. Fraction of replicates assigned to MoAs (A – Lasso, B – Random Forest). For each replicate, the MoA with the highest score is determined and the fraction of replicates assigned to a specific MoA is recorded. This provides an alternative view on the same data as Figure 4 but focuses on the decision for a specific MoA instead of absolute prediction scores.

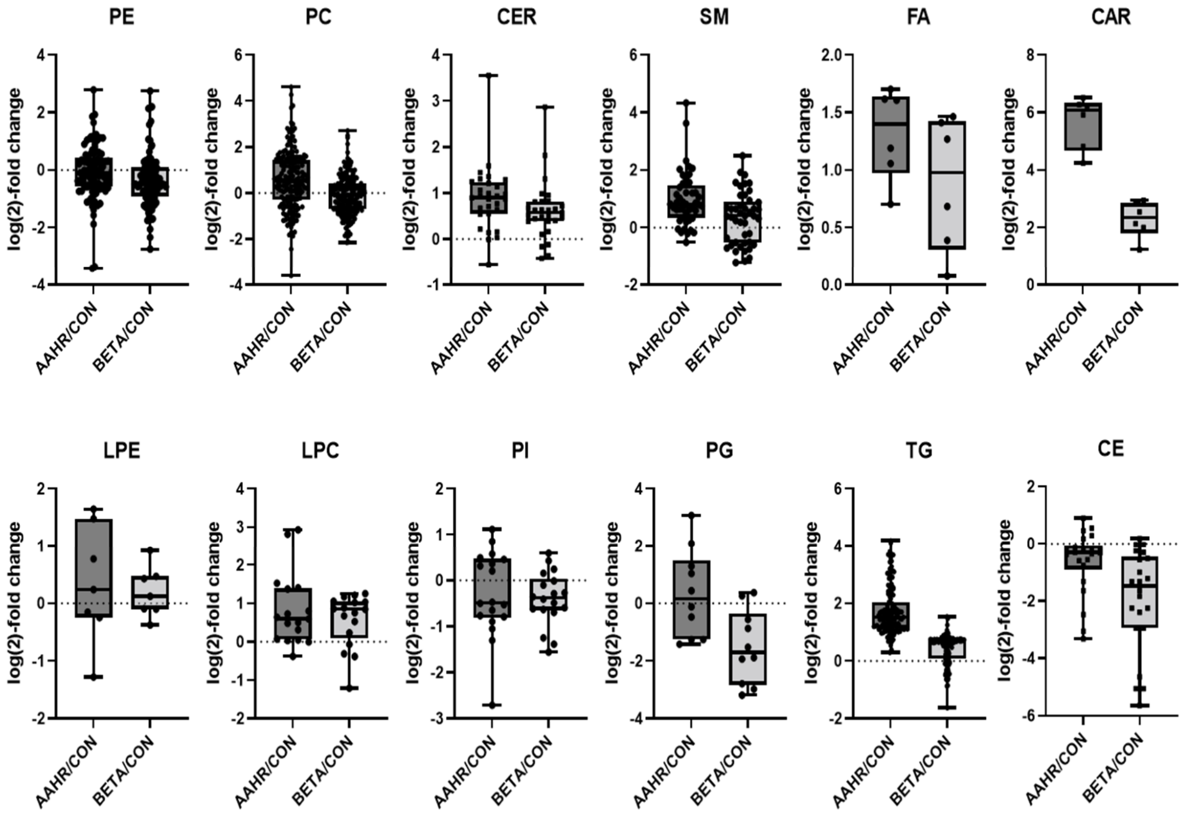

Fig. S8. The ratio of lipid levels of PC-3 cells treated for 48 h with AAHR (285 nM) or BETA (20 μM) versus DMSO controls. Each dot represents an individual lipid. All values are normalized to cell number and represent the average of five independent experiments per annotated lipid. Annotations of individual lipids and their peak heights are presented in Table S5.
PE – diacylglycerophosphoethanolamines, PC – diacylglycerophosphocholines, CER – ceramide, SM – sphingomyolin, FA – free fatty acids, CAR – acylcarnitines, LPE – monoacylglycerophosphoethanolamines, LPC - monoacylglycerophosphocholines, PI – phosphoinositides, PG – phosphoglycerates, TG – triacylglycerides, CE – cholesterol esters.

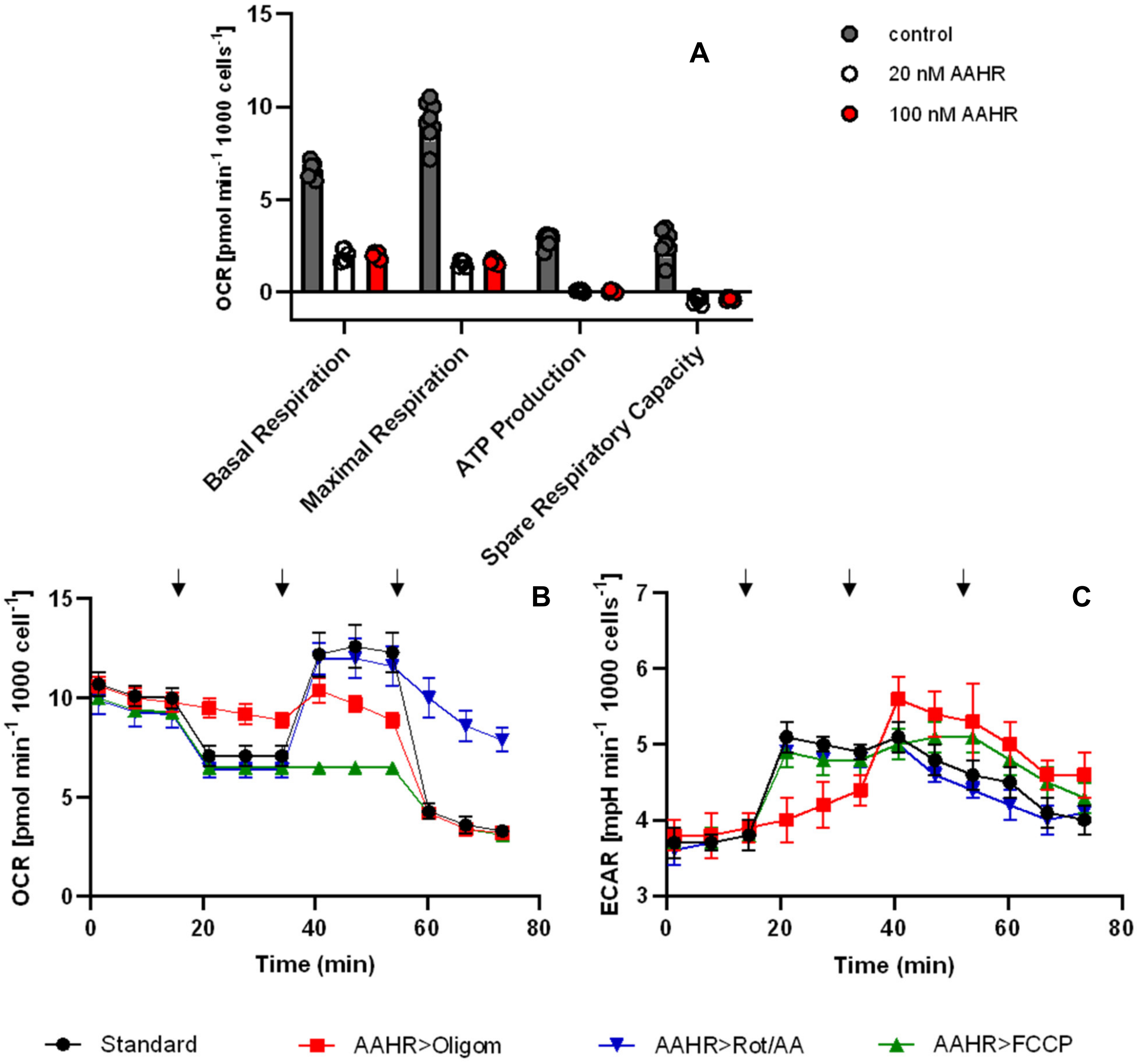

Fig. S9. Analyses of mitochondrial function and ATP production in PC-3 cells.
(A) Parameters obtained from a Seahorse XF Cell Mito Stress Test following a 24 h treatment with AAHR. Basal respiration: oxygen consumption rate (OCR) used to investigate cellular ATP demand resulting from mitochondrial respiration at baseline; Maximal respiration: OCR attained by stimulating the respiratory chain to operate at maximum capacity, shows the maximum rate of respiration the cell can achieve. Spare respiratory capacity indicates the capability of the cell to respond to an energetic demand and how close the cell is to returning to its theoretical maximum. ATP production: oxygen consumption linked cellular ATP production rate.
(B) Patterns of OCR, and **(C)** ECAR (extracellular acidification rate) during the Seahorse XF Cell Mito Stress Test. Arrows indicate the sequential substance injection. In a standard assay, the order of injection is: 1. oligomycin, 2. FCCP, 3. rotenone/antimycin A. In the modified assays, one of the substances was replaced by AAHR, respectively.

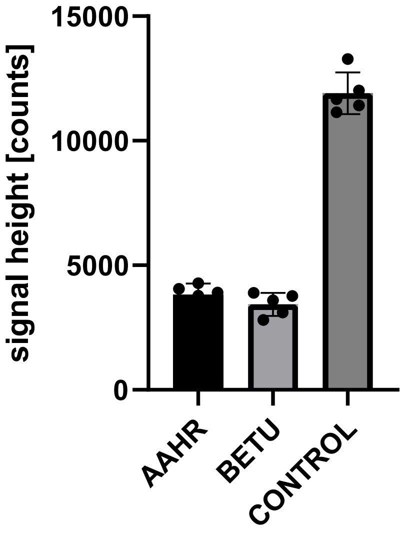

Fig. S10. Analyses of cellular coenzyme Q levels after 48 h treatment with AAHR (285 nM), BETA (20 μM) and DMSO (control).

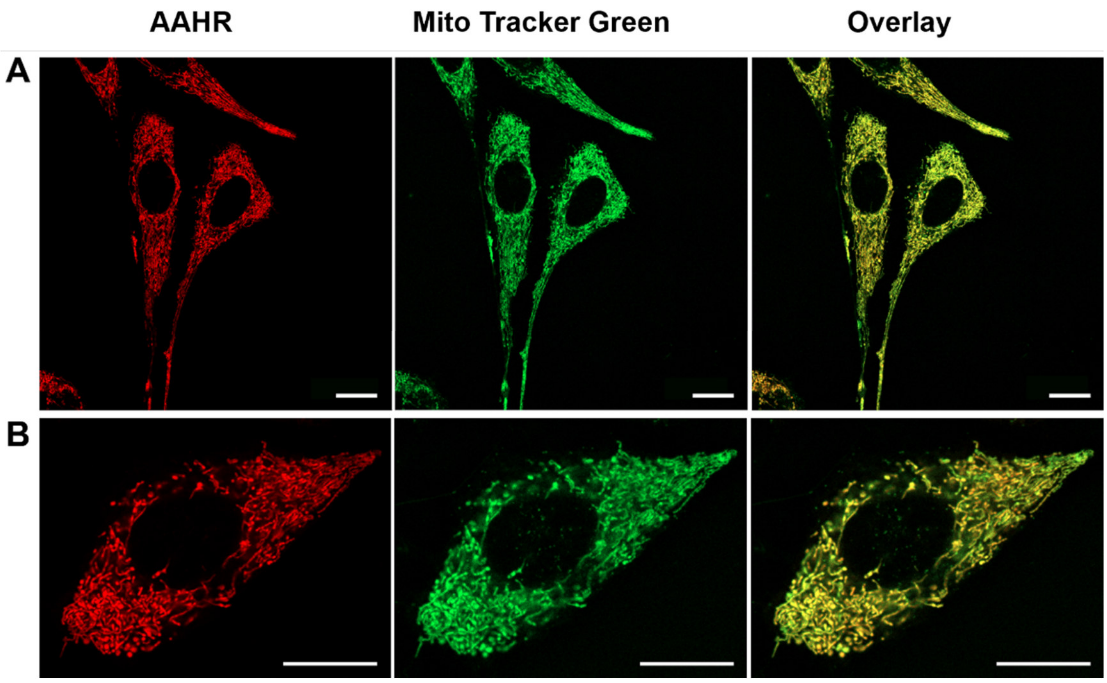

Fig. S11. AAHR accumulation in the mitochondria of PC-3 cells.
PC-3 cells were treated with AAHR for 4 h followed by counterstaining with MitoTracker^{TM} Green and analysis by performing confocal laser scanning microscopy. **(A)** Overview of a few cells; **(B)** close-up of a single cell. The fluorescence of AAHR and MitoTracker^{TM} Green is depicted in red and green, respectively. The overlay shows the co-localization of both AAHR and MitoTracker^{TM} Green. Pearson's Correlation Coefficient was calculated using eight micrographs and revealed 0.8558±0.058. Bars represent 20 μm.

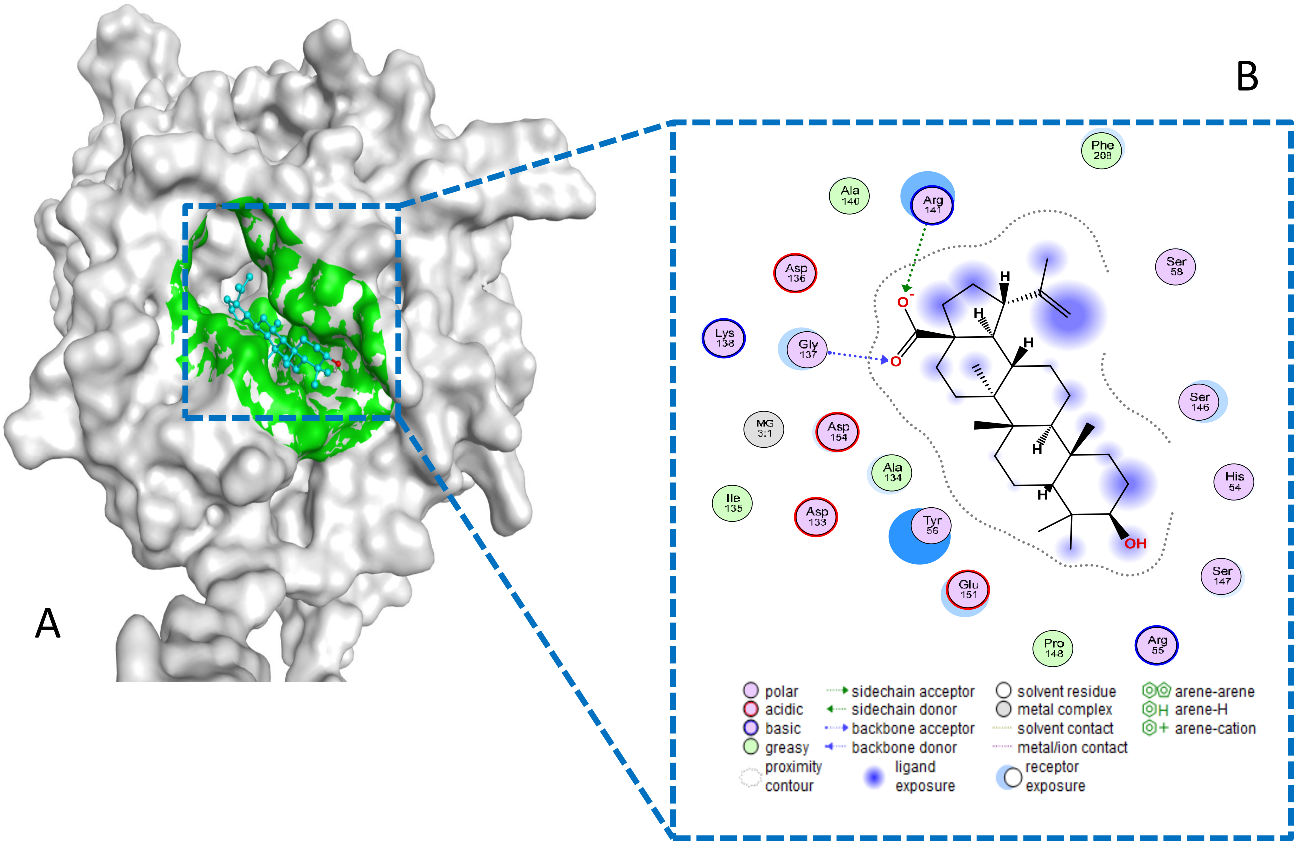

Fig. S12. Molecular docking of pentacyclic triterpenes with CEPT1 and CPT1. **(A)** Molecular surface of CEPT1 with docked BETA as ligand. The binding site is represented in green. **(B)** 2D-interactions showing that the carboxyl group of acid-based pentacyclic diterpenes exhibits favorable hydrogen bond interactions, while the aromatic part has hydrophobic interactions with non-polar residues.

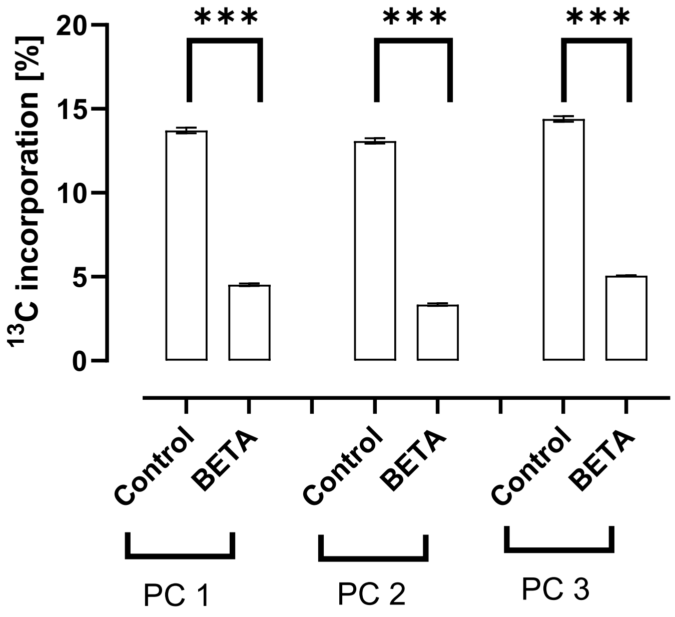

Fig. S13. Comparison of the fractional ^{13}C incorporation in the three most abundant phosphatidylcholines after 48 h treatment with betulinic acid versus untreated controls. PC1: PC 34:1(PC 16:0,18:1); PC2: PC 36:1(PC 18:0,18:1); PC3: PC 32:1(PC 16:0,16:1). Signal intensities are normalized to the cell count. Error bars represent S.D. Significance level 0.001 is based on pairwise t-test with homoscedastic data distribution (n=3).

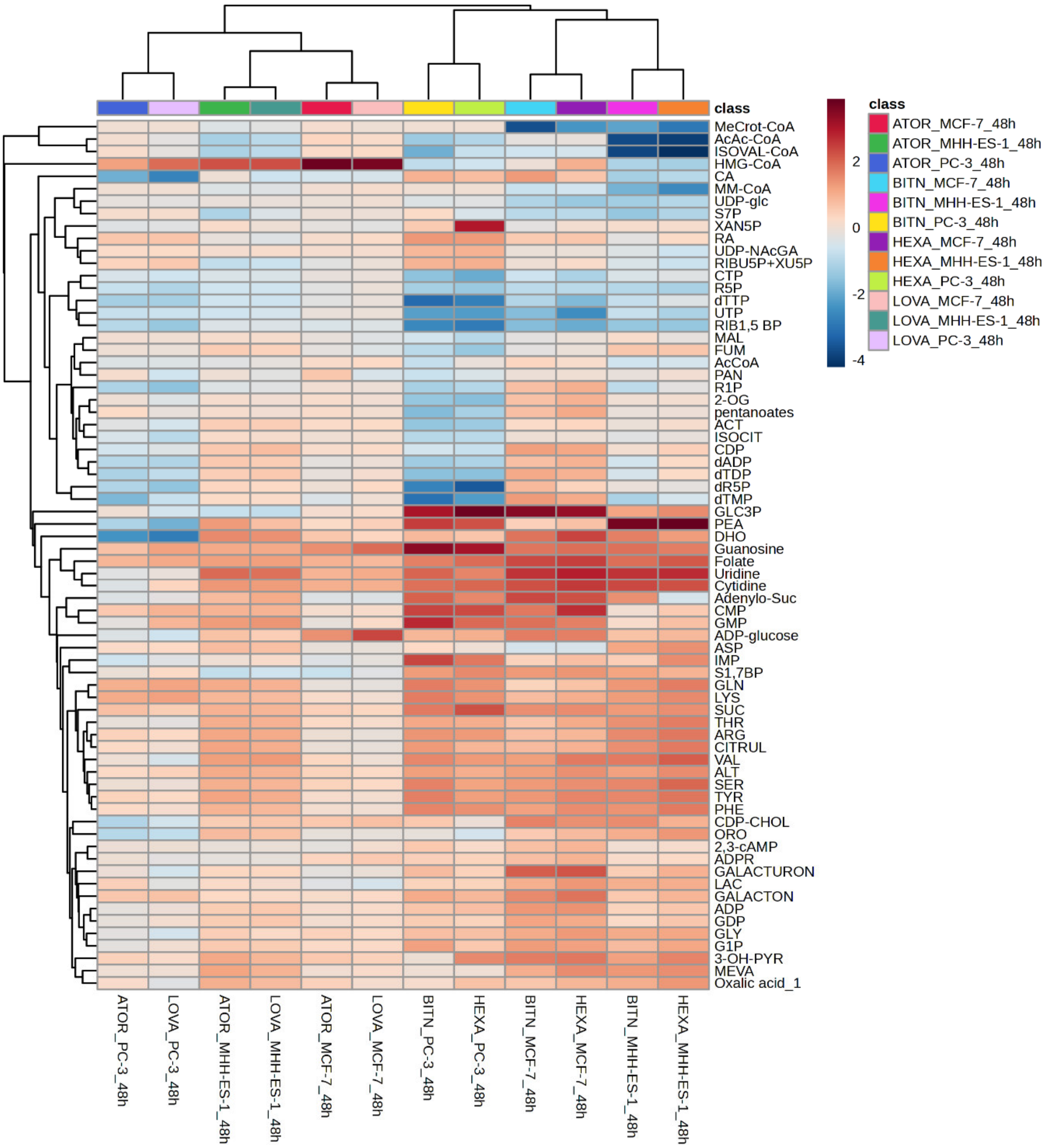

Fig. S14A. Relative abundance of 70 most varying metabolites for the PC-3, MCF-7 and MHH-ES-1 cells treated with four compounds, which target two enzymes: glutamate dehydrogenase (GDH) using HEXA and BITN and HMG-CoA reductase (HMG-CoAr) using the LOVA and ATOR as inhibitors. Data depict average log2-fold changes of cell number-normalized peak areas obtained after 48 h drug treatment (n=6) relative to a vehicle control (n=6). Legends of MoA and compound labels are given in Fig. 1 and Tables S1 and S2.

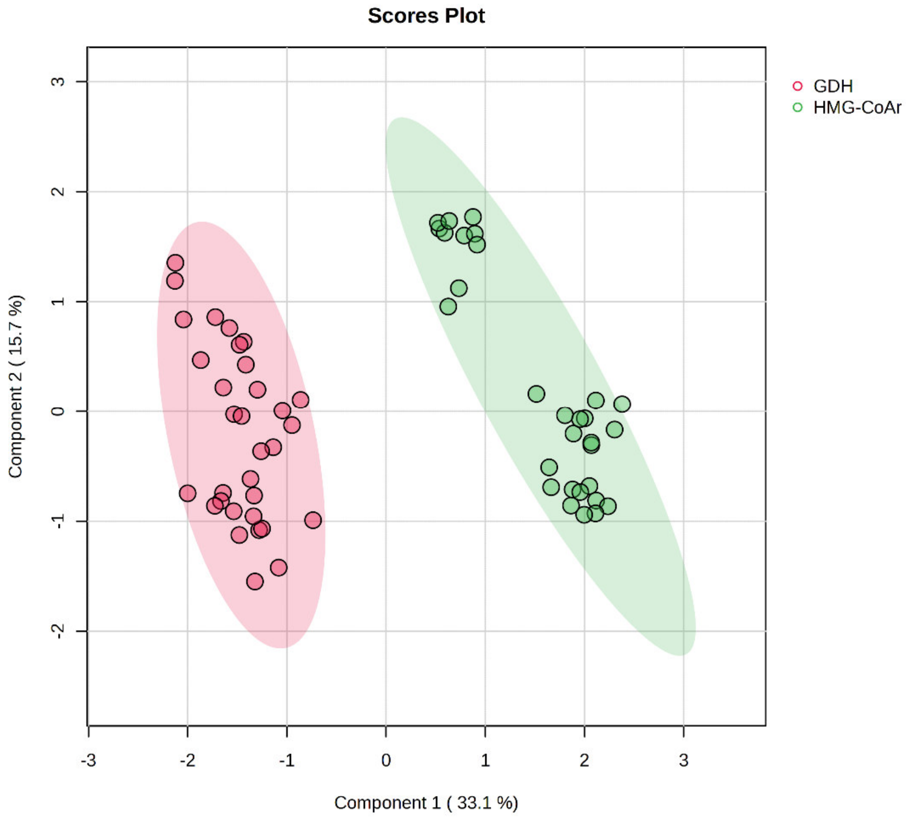

Fig. S14B. Partial Least Squares Discriminant Analysis (PLS-DA) showing scores when hexuplicates of PC-3, MCF-7 and MHH-ES-1 were treated either with statins (HMG-CoAr inhibitors: LOVA, ATOR) or glutamate dehydrogenease inhibitors (BITN, HEXA).

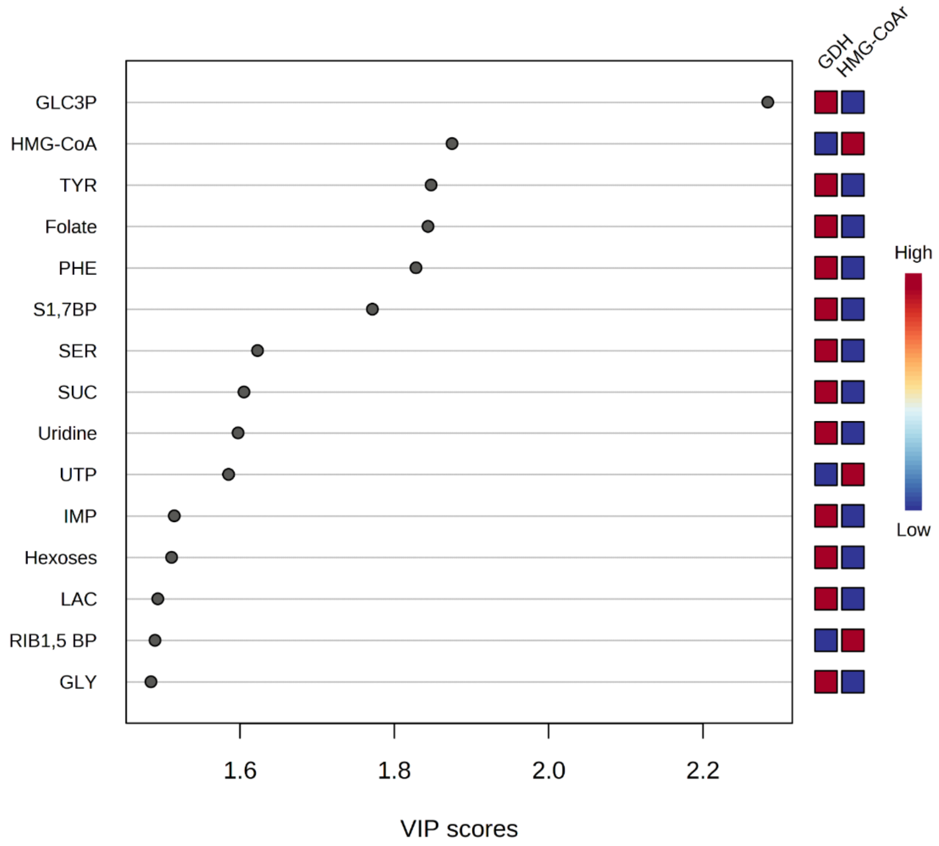

Fig. S14C. Variable of Importance (VIP) scores depicting the 15 most significant metabolites (VIP > 1.2) contributing to the group separations observed in the PLS-DA model (in panel B) differentiating HMG-CoAr inihibition and GDH inhibiton across three cancer cell lines (PC-3, MCF-7 and MHH-ES-1 done using MetaboAnalyst.

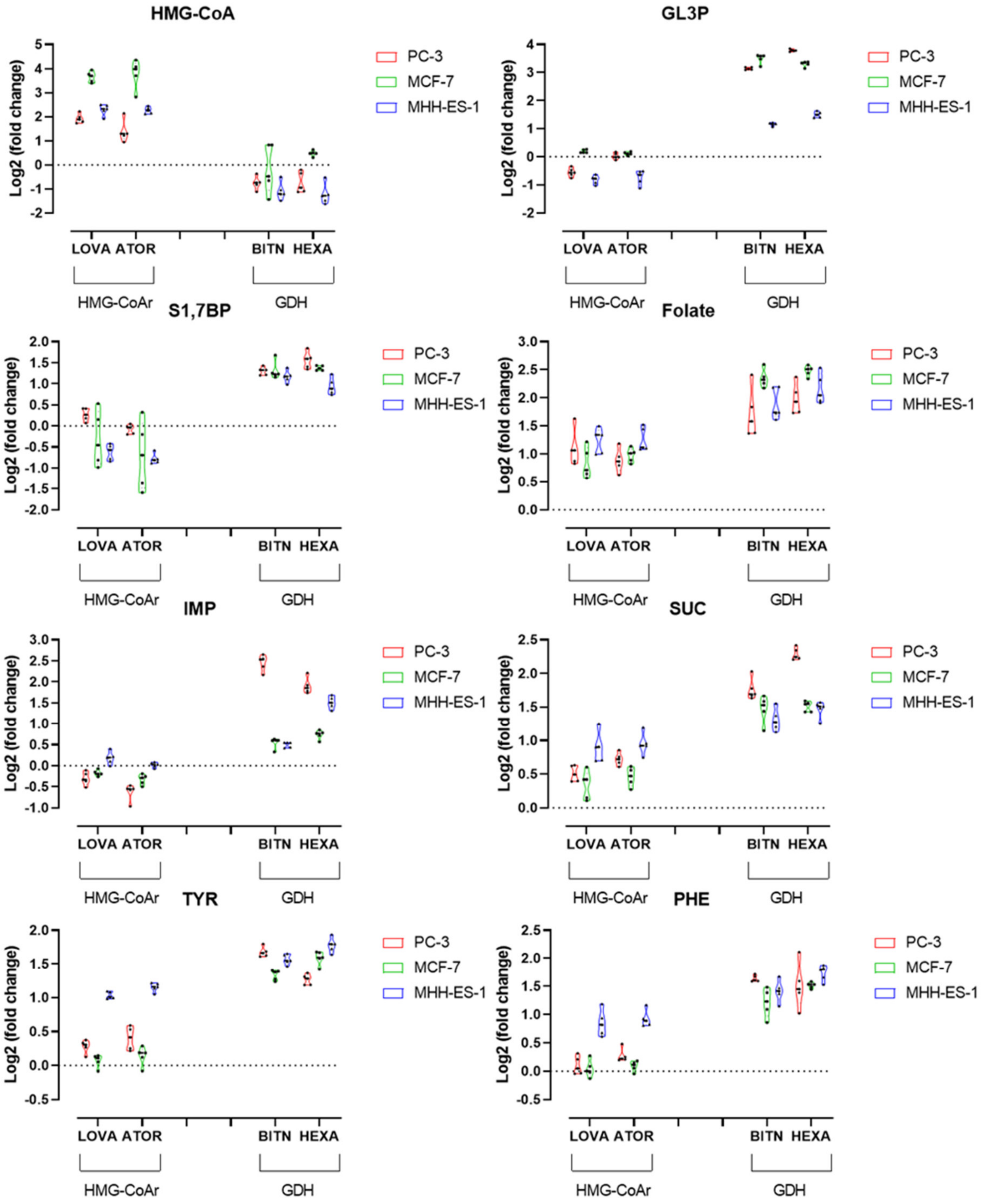

Fig. S14D Selected VIP metabolites of the PLS-DA analysis (in panel B) differentiating HMG-CoAr inihibition and GDH inhibiton across three cancer cell models (PC-3, MCF-7 and MHH-ES-1).

A: Lasso - average

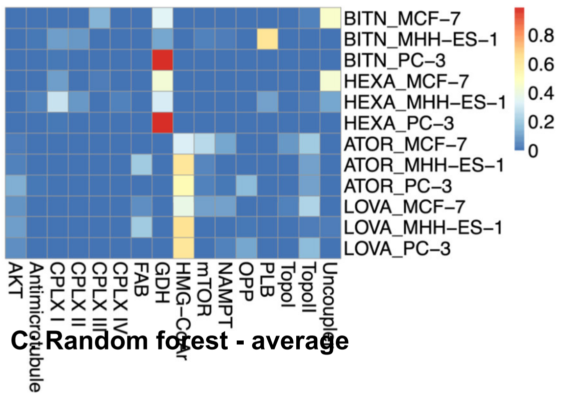

B: Lasso – fraction of replicates

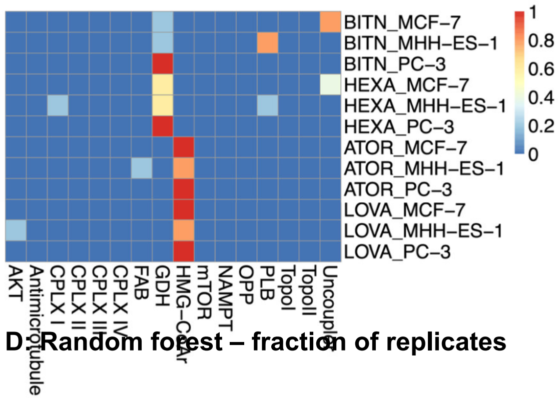

C: Random forest - average

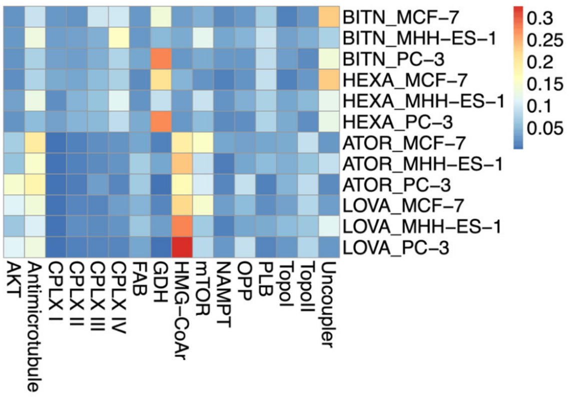

D: Random forest – fraction of replicates

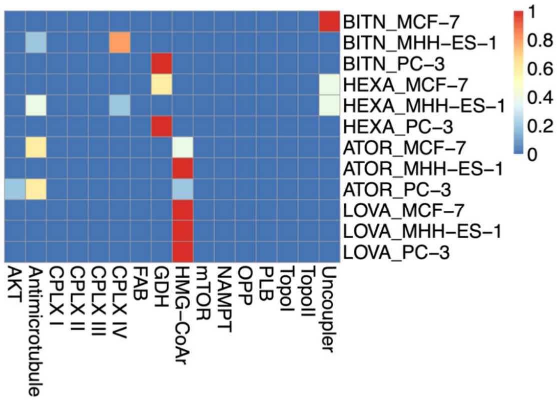

Fig. S14E. Prediction results across cell models for the Lasso (A-B) and Random Forest (C-D) methods. Panels A and C show average prediction scores in analogy to Figure 4D-E, while Panels B and D show the fraction of replicates assigned to a specific MoA in analogy to Figure S12. While the Random Forest classifier widely fails to yield clear prediction scores and classifies several drugs incorrectly, the Lasso method only fails for BITN, but successfully predicts the correct MoA of HEXA, ATOR and LOVA for the majority of replicates in the MCF-7 and MHH-ES-1 cell models.

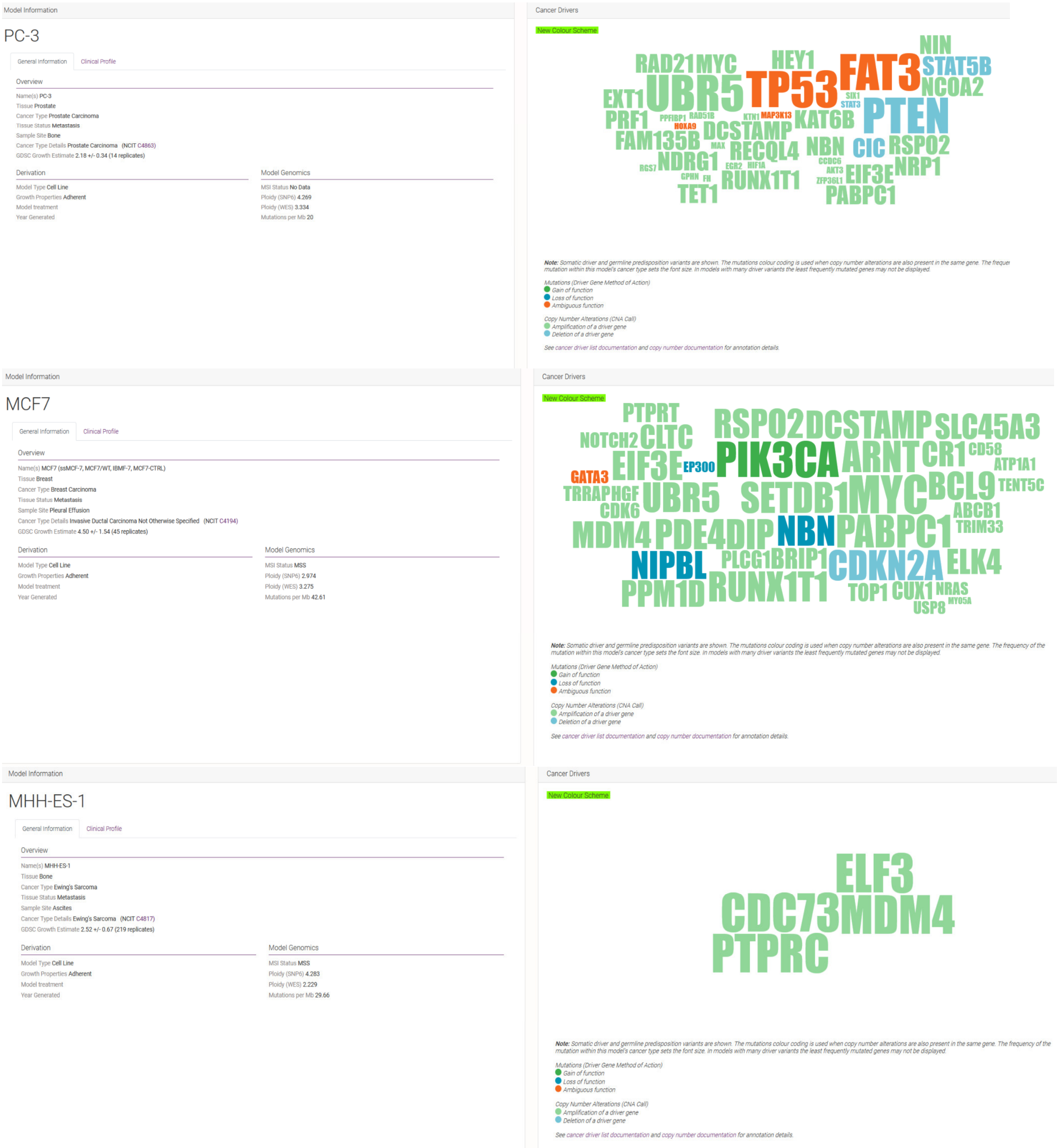

Fig. S15. Cell model passports (https://cellmodelpassports.sanger.ac.uk) showing different mutations in three cancer cell lines (PC-3, MCF-7 and MHH-ES-1)

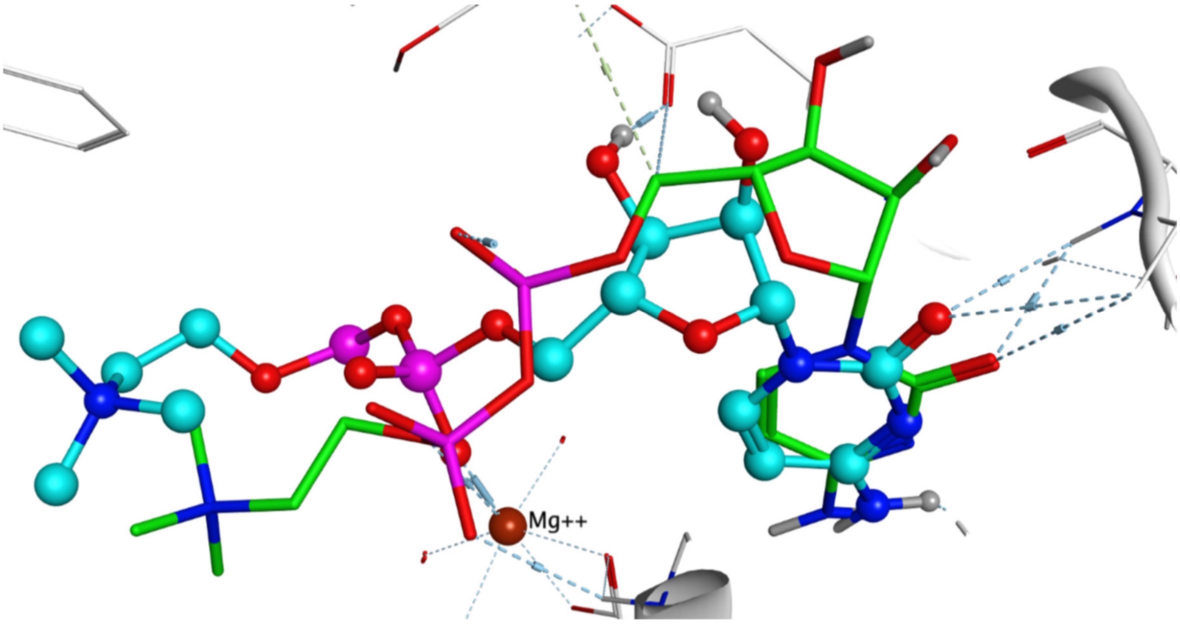

Fig S16. Validation of the molecular docking protocol for ligand binding. The superposition of the ligand (CDP-choline) binding within the Cryo-EM structure of CEPT1 (in cyan) with the docked pose (green), (RMSD of 3.22 Å).
