# Supplementary material for: Advancing Anticancer Drug Discovery: Leveraging Metabolomics and Machine Learning for Mode of Action Prediction by Pattern Recognition
Source: Adv Sci (Weinh). 2024 Oct 21;11(47):2404085. doi: 10.1002/advs.202404085 (PMC11653622; doi:10.1002/advs.202404085)
Supplement: Supplementary file 2 — Supplemental Table 1 [file ADVS-11-2404085-s003.docx]

| Training/ Prediction | Compound | Isomeric SMILES | Abbreviation  (drug) | MoA | Abbreviation  (MOA) | IC_50_ (MTT) | Pathway |
| --- | --- | --- | --- | --- | --- | --- | --- |
| **TRAINING** | 3-Nitropropionic acid | C(C[N+](=O)[O-])C(=O)O | 3-NP | Complex II  Inhibitor (1, 2) | CPLX II | 7.02 mM | Mitochondrial respiration |
|  | Malonic acid | C(C(=O)O)C(=O)O | MALO |  |  | 7.13 mM |  |
|  | Metformin | CN(C(=N)N=C(N)N)C | METF | Complex I  Inhibitor (3, 4) | CPLX I | 16.68 mM |  |
|  | Rotenone | CC(=C)[C@H]1CC2=C(O1)C=CC3=C2O[C@@H]4COC5=CC(=C(C=C5[C@@H]4C3=O)OC)OC | ROTN |  |  | 1.0 µM |  |
|  | Antimycin A | CCCCCC[C@@H]1[C@H]([C@@H](OC(=O)[C@H]([C@H](OC1=O)C)NC(=O)C2=C(C(=CC=C2)NC=O)O)C)OC(=O)CC(C)C | AMYC | Complex III  Inhibitor (4, 5) | CPLX III | 35.6 µM |  |
|  | Atovaquone | OC1=C([C@H]2CC[C@@H](CC2)C2=CC=C(Cl)C=C2)C(=O)C2=CC=CC=C2C1=O | ATOV |  |  | 8.9 µM |  |
|  | Potassium cyanide | [C-]#N.[K+] | CYAN | Complex IV  inhibitor (6) | CPLX IV | 11.6 mM |  |
|  | Sodium azide | [N-]=[N+]=[N-].[Na+] | AZID |  |  | 9.27 mM |  |
|  | 2,4-Dinitrophenol | C1=CC(=C(C=C1[N+](=O)[O-])[N+](=O)[O-])O | 2DNP | Uncoupler (7, 8) | Uncoupler | 0.46 mM |  |
|  | Emodin | CC1=CC2=C(C(=C1)O)C(=O)C3=C(C2=O)C=C(C=C3O)O | EMOD |  |  | 37.0 µM |  |
|  | Carbonyl cyanide m-chlorophenyl hydrazone | C1=CC(=CC(=C1)Cl)NN=C(C#N)C#N | CCCP |  |  | 4.7 µM |  |
|  | Lovastatin | CC[C@H](C)C(=O)O[C@H]1C[C@H](C=C2[C@H]1[C@H]([C@H](C=C2)C)CC[C@@H]3C[C@H](CC(=O)O3)O)C | LOVA | HMG-CoA  reductase  inhibitor (9) | HMG-CoAr | 1.2 µM  12.18 µM*  8.02 µM** | Mevalonate Pathway |
|  | Atorvastatin | CC(C)C1=C(C(=C(N1CC[C@H](C[C@H](CC(=O)O)O)O)C2=CC=C(C=C2)F)C3=CC=CC=C3)C(=O)NC4=CC=CC=C4 | ATOR |  |  | 3.5 µM  24.64 µM*  11.09 µM** |  |
|  | Fluvastatin | CC(C)N1C2=CC=CC=C2C(=C1/C=C/[C@H](C[C@H](CC(=O)O)O)O)C3=CC=C(C=C3)F | FLUV |  |  | 1.0 µM |  |
|  | Paclitaxel | CC1=C2[C@H](C(=O)[C@@]3([C@H](C[C@@H]4[C@]([C@H]3[C@@H]([C@@](C2(C)C)(C[C@@H]1OC(=O)[C@@H]([C@H](C5=CC=CC=C5)NC(=O)C6=CC=CC=C6)O)O)OC(=O)C7=CC=CC=C7)(CO4)OC(=O)C)O)C)OC(=O)C | PTXL | Antimicrotubule (10, 11) | Antimicrotubule | 1.6 nM | Cytoskeleton |
|  | Vincristin | CC[C@@]1(C[C@@H]2C[C@@](C3=C(CCN(C2)C1)C4=CC=CC=C4N3)(C5=C(C=C6C(=C5)[C@]78CCN9[C@H]7[C@@](C=CC9)([C@H]([C@@]([C@@H]8N6C=O)(C(=O)OC)O)OC(=O)C)CC)OC)C(=O)OC)O | VINC |  |  | 0.9 nM |  |
|  | Doxorubicin | C[C@H]1[C@H]([C@H](C[C@@H](O1)O[C@H]2C[C@@](CC3=C2C(=C4C(=C3O)C(=O)C5=C(C4=O)C(=CC=C5)OC)O)(C(=O)CO)O)N)O | DOXO | Topoisomerase II inhibitor (12) | TopoII | 1.2 µM | DNA  replication |
|  | Mitoxantrone | OCCNCCNC1=C2C(=O)C3=C(O)C=CC(O)=C3C(=O)C2=C(NCCNCCO)C=C1 | MITO |  |  | 1.6 µM |  |
|  | Etoposide | C[C@@H]1OC[C@@H]2[C@@H](O1)[C@@H]([C@H]([C@@H](O2)O[C@H]3[C@H]4COC(=O)[C@@H]4[C@@H](C5=CC6=C(C=C35)OCO6)C7=CC(=C(C(=C7)OC)O)OC)O)O | ETOP |  |  | 48.5 µM |  |
|  | Irinotecan | CCC1=C2CN3C(=CC4=C(C3=O)COC(=O)[C@@]4(CC)O)C2=NC5=C1C=C(C=C5)OC(=O)N6CCC(CC6)N7CCCCC7 | IRIN | Topoisomerase I inhibitor (13) | TopoI | 67.4 µM |  |
|  | Camptothecin | CC[C@@]1(C2=C(COC1=O)C(=O)N3CC4=CC5=CC=CC=C5N=C4C3=C2)O | CMPT |  |  | 0.8 µM |  |
|  | Daporinad | C1CN(CCC1CCCCNC(=O)/C=C/C2=CN=CC=C2)C(=O)C3=CC=CC=C3 | FK866 | NAMPT  Inhibitor (14) | NAMPT | 0.3 nM ^#^ | NAD+ salvage |
|  | GMX1778 | C1=CC(=CC=C1OCCCCCCN=C(NC#N)NC2=CC=NC=C2)Cl | GMX |  |  | 0.1 nM ^#^ |  |
|  | GNE617 | C1=CC(=CC=C1CNC(=O)C2=CN3C=CN=C3C=C2)S(=O)(=O)C4=CC(=CC(=C4)F)F | GNE |  |  | 20.0 µM ^#^ |  |
|  | Hexachlorophene | CLC1CC(CL)C(C(C1O)CC1C(O)C(CL)CC(C1CL)CL)CL | HEXA | GDH  Inhibitor (15) | GDH | 10.0 µM  20.72 µM*  10.51 µM** | Glutaminolysis |
|  | Bithionol | CLC1CC(SC2CC(CL)CC(C2O)CL)C(C(C1)CL)O | BITN |  |  | 31.3 µM  22.25 µM*  20.87 µM** |  |
|  | Rapamycin | C[C@@H]1CC[C@H]2C[C@@H](/C(=C/C=C/C=C/[C@H](C[C@H](C(=O)[C@@H]([C@@H](/C(=C/[C@H](C(=O)C[C@H](OC(=O)[C@@H]3CCCCN3C(=O)C(=O)[C@@]1(O2)O)[C@H](C)C[C@@H]4CC[C@H]([C@@H](C4)OC)O)C)/C)O)OC)C)C)/C)OC | RAPA | PI3K/mTOR  Inhibitor (16, 17) | mTOR | 0.05 µM | Signaling/ Protein phosphorylation |
|  | Wortmannin | CC(=O)O[C@@H]1C[C@]2([C@@H](CCC2=O)C3=C1[C@]4([C@H](OC(=O)C5=COC(=C54)C3=O)COC)C)C | WRTN |  |  | 4.1 µM |  |
|  | Alpelisib | CC1=C(SC(=N1)NC(=O)N2CCC[C@H]2C(=O)N)C3=CC(=NC=C3)C(C)(C)C(F)(F)F | ALPL |  |  | 26.1 µM |  |
|  | Perifosine | CCCCCCCCCCCCCCCCCCOP(=O)(OC1CC[N+](CC1)(C)C)[O-] | PRFN | AKT  Inhibitor (18, 19) | AKT | 2.6 µM |  |
|  | Oridonin | CC1(CC[C@@H]([C@]23[C@@H]1[C@@H]([C@]([C@]45[C@H]2CC[C@H]([C@H]4O)C(=C)C5=O)(OC3)O)O)O)C | ORID |  |  | 15.7 µM |  |
|  | 6-Aminonicotinamide | NC(=O)C1CCC(N)NC1 | 6-AN | Inhibitor of  oxidative PPP (20, 21) | OPP | 180.0 µM^#^ | NADPH biosynthesis |
|  | glucose-6-phosphate dehydrogenase (G6PD) inhibitor-1 | C1CCC(=O)C2=CN=C(N=C2C1)NC3=CSC(=C3)C#N | GPDi |  |  | 59.0 µM |  |
|  | Maslinic acid | C[C@@]12CC[C@@H]3[C@@]([C@H]1CC=C4[C@]2(CC[C@@]5([C@H]4CC(CC5)(C)C)C(=O)O)C)(C[C@H]([C@@H](C3(C)C)O)O)C | MASA | Putative CEPT1  Inhibitor (22) | PLB | 57.6 µM | Induce apoptosis |
|  | Betulinic acid | CC(=C)[C@@H]1CC[C@]2([C@H]1[C@H]3CC[C@@H]4[C@]5(CC[C@@H](C([C@@H]5CC[C@]4([C@@]3(CC2)C)C)(C)C)O)C)C(=O)O | BETA |  |  | 19.8 µM |  |
|  | 11-Keto-beta-boswellic acid | C[C@@H]1CC[C@@]2(CC[C@@]3(C(=CC(=O)[C@H]4[C@]3(CC[C@@H]5[C@@]4(CC[C@H]([C@]5(C)C(=O)O)O)C)C)[C@@H]2[C@H]1C)C)C | BOWA |  |  | 76.4 µM |  |
|  | Epigallocatechin gallate | C1[C@H]([C@H](OC2=CC(=CC(=C21)O)O)C3=CC(=C(C(=C3)O)O)O)OC(=O)C4=CC(=C(C(=C4)O)O)O | EGCG | FASN  Inhibitor (23, 24) | FAB | 61.4 µM | Lipid biosynthesis |
|  | Apigenin | OC1CCC(CC1)C1CC(=O)C2C(O1)CC(CC2O)O | APIG |  |  | 22.8 µM |  |
| **Neg. control** | 5-Flurouracil | Fc1c[nH]c(=O)[nH]c1=O. | 5-FU | Thymidylate synthase inhibitor (25) | TS | 100 µM | Nucleotide biosynthesis |
| **PREDICTION** | Breastin |  | BRST | Unknown |  | 1.5 µg/ml |  |
|  | AAHR | C[C@@]12CC[C@]([C@@](COC(C)=O)(C)[C@H]3OC(C)=O)([H])[C@@](C[C@H]3OC(C)=O)(C)[C@@]1([H])CC=C4[C@@]2(C)CC[C@](CC5)(C(N6CCCN(C(C7=CC=CC=C7C(C8=C(C=C(N(CC)CC)C=C8)O9)=C(C=C/%10)C9=CC%10=[N+](CC)\CC)=O)CC6)=O)[C@@]4([H])[C@@H](C)[C@@H]5C.[Cl-] | AAHR |  |  | 285 nM |  |
|  | Cucurbitacin B | CC(=O)OC(C)(C)/C=C/C(=O)[C@@](C)([C@H]1[C@@H](C[C@@]2([C@@]1(CC(=O)[C@@]3([C@H]2CC=C4[C@H]3C[C@@H](C(=O)C4(C)C)O)C)C)C)O)O | QQrB |  |  | 30 nM |  |
|  | Glycyrrhetinic acid | C[C@]12CC[C@](C[C@H]1C3=CC(=O)[C@@H]4[C@]5(CC[C@@H](C([C@@H]5CC[C@]4([C@@]3(CC2)C)C)(C)C)O)C)(C)C(=O)O | GLYA |  |  | 73.1 µM |  |

#: CV results

*: MCF-7 breast cancer cell line

**: MHH-ES1 Ewing’ sarcoma

**References:**

1. Kluckova K, Bezawork-Geleta A, Rohlena J, Dong L, Neuzil J. Mitochondrial complex II, a novel target for anti-cancer agents. Biochim Biophys Acta. 2013;1827(5):552-64.

2. Wojtovich AP, Smith CO, Haynes CM, Nehrke KW, Brookes PS. Physiological consequences of complex II inhibition for aging, disease, and the mKATP channel. Biochim Biophys Acta. 2013;1827(5):598-611.

3. Fontaine E. Metformin-Induced Mitochondrial Complex I Inhibition: Facts, Uncertainties, and Consequences. Front Endocrinol (Lausanne). 2018;9:753.

4. Wang L, Duan Q, Wang T, Ahmed M, Zhang N, Li Y, et al. Mitochondrial Respiratory Chain Inhibitors Involved in ROS Production Induced by Acute High Concentrations of Iodide and the Effects of SOD as a Protective Factor. Oxid Med Cell Longev. 2015;2015:217670.

5. Owens KM, Kulawiec M, Desouki MM, Vanniarajan A, Singh KK. Impaired OXPHOS complex III in breast cancer. PLoS One. 2011;6(8):e23846.

6. Hargreaves IP, Duncan AJ, Wu L, Agrawal A, Land JM, Heales SJ. Inhibition of mitochondrial complex IV leads to secondary loss complex II-III activity: implications for the pathogenesis and treatment of mitochondrial encephalomyopathies. Mitochondrion. 2007;7(4):284-7.

7. Demine S, Renard P, Arnould T. Mitochondrial Uncoupling: A Key Controller of Biological Processes in Physiology and Diseases. Cells. 2019;8(8).

8. Terada H. Uncouplers of oxidative phosphorylation. Environ Health Perspect. 1990;87:213-8.

9. Goulitquer S, Croyal M, Lalande J, Royer AL, Guitton Y, Arzur D, et al. Consequences of blunting the mevalonate pathway in cancer identified by a pluri-omics approach. Cell Death Dis. 2018;9(7):745.

10. Qi C, Wang X, Shen Z, Chen S, Yu H, Williams N, et al. Anti-mitotic chemotherapeutics promote apoptosis through TL1A-activated death receptor 3 in cancer cells. Cell Res. 2018;28(5):544-55.

11. Weaver BA. How Taxol/paclitaxel kills cancer cells. Mol Biol Cell. 2014;25(18):2677-81.

12. Nitiss JL. Targeting DNA topoisomerase II in cancer chemotherapy. Nat Rev Cancer. 2009;9(5):338-50.

13. Talukdar A, Kundu B, Sarkar D, Goon S, Mondal MA. Topoisomerase I inhibitors: Challenges, progress and the road ahead. Eur J Med Chem. 2022;236:114304.

14. Navas LE, Carnero A. Nicotinamide Adenine Dinucleotide (NAD) Metabolism as a Relevant Target in Cancer. Cells. 2022;11(17).

15. Bian Y, Hou W, Chen X, Fang J, Xu N, Ruan BH. Glutamate Dehydrogenase as a Promising Target for Hyperinsulinism Hyperammonemia Syndrome Therapy. Curr Med Chem. 2022;29(15):2652-72.

16. Vasan N, Cantley LC. At a crossroads: how to translate the roles of PI3K in oncogenic and metabolic signalling into improvements in cancer therapy. Nat Rev Clin Oncol. 2022;19(7):471-85.

17. Peng Y, Wang Y, Zhou C, Mei W, Zeng C. PI3K/Akt/mTOR Pathway and Its Role in Cancer Therapeutics: Are We Making Headway? Front Oncol. 2022;12:819128.

18. Gills JJ, Dennis PA. Perifosine: update on a novel Akt inhibitor. Curr Oncol Rep. 2009;11(2):102-10.

19. Song M, Liu X, Liu K, Zhao R, Huang H, Shi Y, et al. Targeting AKT with Oridonin Inhibits Growth of Esophageal Squamous Cell Carcinoma In Vitro and Patient-Derived Xenografts In Vivo. Mol Cancer Ther. 2018;17(7):1540-53.

20. Tyson RL, Perron J, Sutherland GR. 6-Aminonicotinamide inhibition of the pentose phosphate pathway in rat neocortex. Neuroreport. 2000;11(9):1845-8.

21. Ghergurovich JM, Garcia-Canaveras JC, Wang J, Schmidt E, Zhang Z, TeSlaa T, et al. A small molecule G6PD inhibitor reveals immune dependence on pentose phosphate pathway. Nat Chem Biol. 2020;16(7):731-9.

22. Yadav VR, Prasad S, Sung B, Kannappan R, Aggarwal BB. Targeting inflammatory pathways by triterpenoids for prevention and treatment of cancer. Toxins (Basel). 2010;2(10):2428-66.

23. Pan MH, Lin CC, Lin JK, Chen WJ. Tea polyphenol (-)-epigallocatechin 3-gallate suppresses heregulin-beta1-induced fatty acid synthase expression in human breast cancer cells by inhibiting phosphatidylinositol 3-kinase/Akt and mitogen-activated protein kinase cascade signaling. J Agric Food Chem. 2007;55(13):5030-7.

24. Brusselmans K, Vrolix R, Verhoeven G, Swinnen JV. Induction of cancer cell apoptosis by flavonoids is associated with their ability to inhibit fatty acid synthase activity. J Biol Chem. 2005;280(7):5636-45.

25. Schmidt DR, Patel R, Kirsch DG, Lewis CA, Vander Heiden MG, Locasale JW. Metabolomics in cancer research and emerging applications in clinical oncology. Ca-Cancer J Clin. 2021;71(4):333-58.
