# Supplementary material for: Advancing Anticancer Drug Discovery: Leveraging Metabolomics and Machine Learning for Mode of Action Prediction by Pattern Recognition
Source: Adv Sci (Weinh). 2024 Oct 21;11(47):2404085. doi: 10.1002/advs.202404085 (PMC11653622; doi:10.1002/advs.202404085)
Supplement: Supplementary file 7 — Supplemental Table 6 [file ADVS-11-2404085-s001.docx]

**Table S4.** Binding affinity of triterpenoid ligands to the CEPT1 and CPT1 in comparison to the native substrate CDP-Choline

|  | [kcal mol^-1^] | |
| --- | --- | --- |
|  | **CEPT1** | **CPT1** |
| Betulinic acid | -8.33 | -7.31 |
| 11-Keto-beta-boswellic acid | -7.3 | -6.17 |
| Glycyrrhetinic acid | -7.6 | -7.68 |
| Maslinic acid | -7.4 | -6.13 |
| Ursolic acid | -8.66 | -7.44 |
| CDP-Choline | -7.14 | -8.33 |
